# Comparative Analysis of Label-Free and 8-Plex iTRAQ Approach for Quantitative Tissue Proteomic Analysis

**DOI:** 10.1371/journal.pone.0137048

**Published:** 2015-09-02

**Authors:** Agnieszka Latosinska, Konstantinos Vougas, Manousos Makridakis, Julie Klein, William Mullen, Mahmoud Abbas, Konstantinos Stravodimos, Ioannis Katafigiotis, Axel S. Merseburger, Jerome Zoidakis, Harald Mischak, Antonia Vlahou, Vera Jankowski

**Affiliations:** 1 Biotechnology Division, Biomedical Research Foundation of the Academy of Athens, Athens, Greece; 2 Charité-Universitätsmedizin Berlin, Berlin, Germany; 3 Institut National de la Santé et de la Recherche Médicale (INSERM), U1048, Institute of Cardiovascular and Metabolic Diseases, Toulouse, France; 4 Université Toulouse III Paul-Sabatier, Toulouse, France; 5 BHF Glasgow Cardiovascular Research Centre, University of Glasgow, Glasgow, United Kingdom; 6 Department of Pathology, Hannover Medical School, Hannover, Germany; 7 Department of Urology, Medical School of Athens, Laikon Hospital, Athens, Greece; 8 Department of Urology, University of Lübeck, Lübeck, Germany; 9 Mosaiques Diagnostics GmbH, Hannover, Germany; 10 RWTH-Aachen, Institute for Molecular Cardiovascular Research (IMCAR), Aachen, Germany; UGent / VIB, BELGIUM

## Abstract

High resolution proteomics approaches have been successfully utilized for the comprehensive characterization of the cell proteome. However, in the case of quantitative proteomics an open question still remains, which quantification strategy is best suited for identification of biologically relevant changes, especially in clinical specimens. In this study, a thorough comparison of a label-free approach (intensity-based) and 8-plex iTRAQ was conducted as applied to the analysis of tumor tissue samples from non-muscle invasive and muscle-invasive bladder cancer. For the latter, two acquisition strategies were tested including analysis of unfractionated and fractioned iTRAQ-labeled peptides. To reduce variability, aliquots of the same protein extract were used as starting material, whereas to obtain representative results per method further sample processing and MS analysis were conducted according to routinely applied protocols. Considering only multiple-peptide identifications, LC-MS/MS analysis resulted in the identification of 910, 1092 and 332 proteins by label-free, fractionated and unfractionated iTRAQ, respectively. The label-free strategy provided higher protein sequence coverage compared to both iTRAQ experiments. Even though pre-fraction of the iTRAQ labeled peptides allowed for a higher number of identifications, this was not accompanied by a respective increase in the number of differentially expressed changes detected. Validity of the proteomics output related to protein identification and differential expression was determined by comparison to existing data in the field (Protein Atlas and published data on the disease). All methods predicted changes which to a large extent agreed with published data, with label-free providing a higher number of significant changes than iTRAQ. Conclusively, both label-free and iTRAQ (when combined to peptide fractionation) provide high proteome coverage and apparently valid predictions in terms of differential expression, nevertheless label-free provides higher sequence coverage and ultimately detects a higher number of differentially expressed proteins. The risk for receiving false associations still exists, particularly when analyzing highly heterogeneous biological samples, raising the need for the analysis of higher sample numbers and/or application of adjustment for multiple testing.

## Introduction

Application of mass spectrometry-based quantitative approaches has largely contributed to the emerging role of proteomics [1]. Quantitative analysis has been widely applied in various proteomics fields such as a) clinical proteomics [2, 3], b) subcellular proteomics [4, 5] or c) interaction proteomics [6, 7]. Moreover, high-resolution, comparative proteomic studies have led to progress in system biology analysis, particularly in the context of elucidation of the mechanisms underlying pathophysiology of various diseases [8].

Currently, two main types of relative quantification strategies for MS-based proteomics analysis exist: a) label-based and b) label-free (LFQ) MS-based approaches [9]. In the label-based approach, the quantification relies on the introduction of stable isotopes. Depending on the methods for isotope incorporation into the peptides/proteins, several labeling protocols have been developed including a) metabolic labeling (stable isotope labeling of amino acids in cell culture), b) chemical labeling (isotope-coded affinity tag, isobaric tag for relative and absolute quantification (iTRAQ), tandem mass tag (TMT)), c) enzymatic labeling (oxygen isotope (^18^O)) or d) external addition of the labeled synthetic peptides [9]. Label-based methods allow for the simultaneous analysis of multiple samples in a single MS run (multiplexing), resulting in reduced analytical variability. This is particularly relevant for the application of TMT and iTRAQ labeling, since up to eight (for iTRAQ) [10] or ten (for TMT) [11] samples can be analyzed simultaneously during a single experiment. In these cases, due to the isobaric nature of labels, labeled peptides appear as a single peak in the full MS scan. However, upon peptide fragmentation at the MS/MS level, the isotope-containing reporter ions are released and distinguished according to their masses based on the label composition.

On the other hand, the label-free approach does not utilize stable isotopes. In this case, the quantification is based on spectral counting and intensity-based measurements. In the former method, quantification occurs at the MS/MS level utilizing the number of fragmentation spectra assigned to peptides that belong to a particular protein. On the contrary, the intensity-based quantification method is applicable at the MS1 level and the quantification is based on the estimated area under the curve from the extracted ion chromatogram [9].

Both, iTRAQ and label-free quantification have been widely applied in proteomic research. Up to date, several studies have been published in order to evaluate their analytical performance including precision, accuracy of quantification, protein sequence coverage and quantification reproducibility [12–16]. In a few studies, an additional effort was made to evaluate the biological significance of the findings. These studies included evaluation of a) two *Chlamydomonas reinharditii* strains in the context of biofuel production [16], b) *Methylocella silverstris* bacterium cultured under various conditions [13] and c) adenovirus infection of human lung cells [15]. In the aforementioned studies, functional analysis of differentially expressed proteins identified in label-free and iTRAQ revealed the de-regulation of proteins associated with the studied process [13, 15, 16]. However, the contribution of the de-regulated proteins to particular biological process varies between both approaches [16], likely as a result of the different analytical performance of both quantification strategies. Along the same lines, a comprehensive comparison of the two methods, as applied in the analysis of complex biological samples (such as tumors) has not been reported yet. Both strategies are advocated and might be used as complementary approaches [17, 18]. Importantly, performance achieved during the analysis of cell lines or bacterial strains (as has been reported so far) may not be representative when the biological variability and/or complexity of samples is high. Based on the above, knowledge on the performance of these quantification strategies would provide valuable guidance on which method to use when dealing with complex and heterogeneous material such as clinical samples.

In this manuscript, we describe a side-by-side comparison of the label-free and label-based (8-plex iTRAQ) methods, with the latter also preceded by an additional fractionation step. The central goal was to provide recommendations on which approach to use when investigating protein differential expression in samples typically used in clinical proteomics. In the presented study, bladder cancer (BCa) tissue specimens representing two different tumor stages (non-muscle invasive vs. muscle invasive) were evaluated. Specifically, the number of identified proteins, their sequence coverage, consistency of reported changes and reliability of findings as defined by agreement with existing transcriptomics data were assessed. To reduce quantification bias, we attempted to unify the sampling process by utilizing aliquots of the same tissue extracts to obtain as representative as possible results per method. Sample processing and analysis by mass spectrometry were performed according to regularly used/optimized protocols per method.

## Materials and Methods

### Clinical samples

Bladder cancer tissue specimens were collected from patients undergoing transurethral resection of bladder cancer in medical centers in Greece (Laikon Hospital, Athens) and Germany (Department of Urology and Urological Oncology, Hannover Medicine School). The studies were approved by the respective local ethics committees (for Athens Ε.S 618–2012 and for Hannover 614–2009) and all individuals gave written informed consent. Samples from tumor tissue from 8 patients were employed for the analysis including non-muscle invasive (stage pTa, n = 4) and muscle invasive bladder cancer cases (stage pT2+, n = 4). Tumor stage was determined according to TNM classification system [19].

### Sample preparation

Approximately 20 mg of bladder cancer tissue was homogenized in 150 μL of lysis buffer (4% SDS, 0.1M DTE, 0.1M Tris-HCl pH 7.6) using blade homogenizer (three cycles of 30 – 40s) followed by sonication (15 s per sample). This protein extraction protocol was selected following preliminary experiments testing the performance of different homogenization means such as homogenization by using liquid nitrogen, Potter homogenizer or ultrasonication (data not shown). Undissolved materials were removed by centrifugation at 13000 rpm for 10 min. Protein concentration was determined by the Bradford assay (BioRad) and protein extracts were processed using the FASP [20], separately for LFQ and iTRAQ experiments.

#### Label free analysis

Equal amount of protein (200 μg) per sample prepared as described above was first subjected to buffer exchange in Amicon Ultra Centrifugal filter devices (0.5 mL, 30 kDa MWCO, Millipore) at 13 000 rpm for 15 min at room temperature. The protein extract was mixed with 200 μL of urea buffer (8M urea in 0.1M Tris-HCl pH 8.5) and centrifugal concentration was performed. The concentrate was then diluted with urea buffer and centrifugation was repeated. Subsequently, alkylation of proteins was performed by adding 100 μL of 0.1M iodoacetamide in urea buffer followed by 20 min incubation in the dark. Samples were centrifuged at 13 000 rpm for 10 min. Additional series of washes were conducted with urea (twice) and ABC buffer (50 mM NH_4_HCO_3_ pH 8, twice). Overnight digestion was performed by adding 2 μg of trypsin (stock solution of 500 ng/μL) in 40 μL of ABC (trypsin to protein ratio 1:100). Peptides were eluted by centrifugation followed by washing with 40 μL of 50 mM NH_4_HCO_3_. Afterwards, samples were lyophilized.

#### 8-plex iTRAQ labeling

100 μg of protein extract was processed by FASP as described above with the following modifications a) 50 mM trietylamonium bicarbonate (TEAB) was used instead of ABC buffer, b) 1 μg of trypsin was added in 20 μL of 50 mM TEAB and c) peptides were eluted with 20 μL of 50 mM TEAB. Tryptic digest peptides were labeled using the 8-plex iTRAQ Reagent kit (AB Sciex) according to manufacturer instructions. Samples from non-invasive tumor tissue (pTa stage) were labeled using 113–116 tags, whereas for the invasive tumors (pT2+) 117–119 and 121 tags were used. Subsequently, 8 individual samples were mixed and lyophilized to dryness. To remove excess of the iTRAQ reagents, peptides were re-suspended in 0.1% formic acid and 80 μg were purified using Pierce C18 Tips, 100μL bed (Thermo Scientific) according to manufacturer instructions. As an alternative approach, a high pH reverse phase chromatography on a Dionex P680 HPLC system was applied to purify and pre-fractionate the remaining peptide mixture (∼ 700 μg). Labeled peptides were lyophilized and redissolved in 250 μL of high pH buffer (0.05% NH_4_OH, pH 9–9.5) by sonication in a water bath. The solution was filtered using syringe driven filter unit (0.22 μM PVDF). After loading of 200 μL onto an XBridge 4.6 x 150 mm C18 column (BEH Technology) at flow rate of 0.4 mL/min in 0.05% NH_4_OH, the sample was eluted with a gradient of solvent A: 0.05% NH_4_OH in water versus solvent B: 0.05% NH_4_OH in 100% acetonitrile starting at 5% B for 15 min, then to 35%B at 25 min then to 80% B at 30 min followed by 5 min rinsing at 80% B. In total, 5 fractions of 1.2 mL were collected starting from 21 min up to 35 min of the gradient. Prior to the LC-MS/MS analysis, 3 of these fractions with the lowest peptide content (1, 4 and 5) were pooled.

### LC-MS/MS analysis

10 μg of protein digest were loaded onto a Dionex Ultimate 3000 RSLS nano flow system (Dionex, Camberly UK). After loading onto a Dionex 0.1×20 mm 5 μm C18 nano trap column at a flow rate of 5 μl/min in 0.1% formic acid and 2% acetonitrile, samples were applied onto an Acclaim PepMap C18 nano column 75 μm×50 cm, 2 μm 100 Å at a flow rate of 0.3 μl/min. The trap and nano flow column were maintained at 35°C. The samples were eluted with a gradient of solvent A: 0.1% formic acid versus solvent B: 80% acetonitrile starting at 1% B for 5 min rising to 5% B at 10 min then to 25% B at 360 min and 65%B at 480 min.

The eluent was ionized using a Proxeon nano spray ESI source operating in positive ion mode into an Orbitrap Velos FTMS (Thermo Finnigan, Bremen, Germany). Ionization voltage was 2.6 kV and the capillary temperature was 200°C. The mass spectrometer was operated in MS/MS mode scanning from 380 to 2000 m/z. The top 20 multiply charged ions were selected from each scan for MS/MS analysis using CID at 40% collision energy. The resolution in MS1 was 60,000 and 7,500 at m/z 400 for CID in MS2. For the iTRAQ samples, the top 20 multiply charged ions were selected from each scan for MS/MS analysis using HCD at 45% collision energy. AGC settings were 1,000,000 for full scan in the FTMS and 200,000 for MSn. Resolution in MS2 at m/z 115 was 16,300. Dynamic exclusion was enabled with a repeat count of 1, exclusion duration of 30 seconds.

### Data processing

The processing of the individual raw MS data files was conducted using the commercially available software Proteome Discoverer v. 1.4.0.288 (Thermo Scientific). An event detection node was used at a setting of 2 ppm along with the precursor ion peak detector node. Database search was carried out against Human Swiss-Prot Database (30/10/2013) [21, 22] containing only the canonical sequences with 20 277 entries using the Sequest search engine [23] implemented in Proteome Discoverer. The following search parameters were applied: a) precursor mass tolerance 10 ppm, b) fragment mass tolerance: 0.8 Da and 0.05 Da for label-free and iTRAQ experiments, respectively, c) fixed modification: carbamidomethylation of cysteine (C) and additionally for the labeling experiment an iTRAQ modification of N-terminus and lysine residues were added, d) variable modification: oxidation of methionine (M) and in the case of iTRAQ, the iTRAQ modification on tyrosine (Y) was added, e) allowing one missed cleavage site. The false discovery rate evaluation was performed by using the Percolator node [24] (Proteome Discoverer 1.4). To verify labeling efficiency, an additional search was performed by setting the iTRAQ 8-plex labels as variable modifications on N-terminus and Lysine (K). In parallel, the prevalence of the modifications (including oxidation, chemically induced cysteine modification, chemical and posttranslational modifications) was evaluated by using Preview™ node (v2.6.46, Protein Metrics Inc.) [25] incorporated in the Proteome Discoverer workflow. To this end, a search was performed for the selected data files from each experimental approach incorporating the modifications indicated above. The mass spectrometry proteomics data have been deposited to the ProteomeXchange Consortium [26] via the PRIDE partner repository with the dataset identifier PXD002170.

### Protein Identification

The same selection criteria were applied for protein identification in both approaches. Identified peptides were initially filtered requiring mass deviation below 5 ppm between experimental and theoretical mass, false discovery rate below 1% (assigned in Proteome Discoverer as high confidence peptides) and peptide rank up to 5. Peptides were excluded if they contained an unknown amino acid (X) in the sequence or if the protein accession could not be mapped. In the case of the label-free approach, the list of the non-redundant peptides for the entire experiment was then generated, based on the individual datasets (due to its multiplexity nature, merging was not required in the case of iTRAQ). During the merging of the individual datasets from the label-free experiment, only peptides with an FDR<1% were included. FDR level was not assessed again after merging of the data. If sequences with identical number of modifications, although in different position, were reported, only one sequence was retained. For each spectrum (as defined by the same m/z and retention time), the best candidate sequence was defined based on the relative number of sequence identifications per sample (e.g. the sequence with the highest number of identifications was maintained). The confidence in the interpretation (based on the XCorr) was taken into consideration in cases where the same number of sequence identifications was reported. Additionally, only peptides consistently reported in more than 75% of the samples (at least in one group: pTa and/or pT2+) were considered as credible. Subsequently, peptides were assigned to the protein according to the Occam Razor principal [27]. All peptides derived from keratins were excluded as probable contaminations, and were not taken into consideration during the subsequent analysis. Only proteins identified based on ≥2 peptides were considered for further comparative analysis.

### Relative Quantification

#### Label-free quantification

The peak area-based quantification uses precursor ions to assess the relative abundance of identified proteins in the label-free data. For each precursor ion, peak area (i.e. area under the curve) is calculated from the extracted ion chromatogram during data processing in Proteome Discoverer by using the Precursor Ions Area Detector node. For the sequences for which no peptide area could be integrated by Proteome Discoverer (version 1.4; this is a well-known, but not yet corrected problem of this software), the absent values were replaced with the mean area values calculated in that group (pTa or pT2+). When the peptide was not identified in the particular sample, the missing values were replaced with zero. Part per million (ppm)-normalization was conducted for the selected peptides according to the following formula: Normalized peak area = (Peptide peak area/Total peak area)×10^6^. Protein abundance in each sample was calculated as the sum of all normalized peptide areas for a given protein. Peptides matching to multiple protein IDs were included only for the quantification of the one protein indicated by the Occam Razor rule [27]. The mean protein abundance per groups was then calculated and the average values were log2 transformed. The log2 ratio was then calculated by the subtraction of the log2 transformed mean value obtained for case and controls [ ${\text{log}}_{2}\frac{case}{control}=Log_{2}Avg.Cases-Log_{2}Avg.Controls$ ].

#### Label-based quantification

All quantification steps were performed using the Proteome Discoverer Software (version 1.4). The 8-plex iTRAQ quantification was performed based on the reporter ion intensities detected by the Reporter Ions Quantifier Node in Proteome Discoverer. The reporter ion intensities were corrected for the isotopic impurities using reporter ion isotopic distribution (S1 Table). When the individual reporter intensities were 0 (the reporter, or mass, tags are missing in the quantification spectrum), the minimal reported intensity was assigned to the respective peptide. To provide an accurate quantification of proteins, only peptide spectrum matches with co-isolation interference below 30% were included in the analysis [28]. Subsequently, for each distinct peptide the abundance was calculated as the median of reporter ions from all matching spectra, since median is more resistant to outliers. Spectra were grouped based on mass and sequence, without taking into consideration the peptide charge. In the case of modifications, the peptides were considered as distinct when modifications were different. The reporter ion intensities for each individual peptide were represented as a ratio of the particular reporter ion to the sum of all reporter (as in the case of Libra implemented in Trans Proteomic Pipeline Software [29]). To account for experimental biases (e.g. unequal loading), the quantification values for each channel were balanced to be equal to 12.5%, which corresponds to the contribution of 1 out of 8 labels for quantification. This is based on the assumption that the reporter ions are ionized with the same efficiency and in the case of equal loading comparable total intensity of reporter ions should be obtained for each label. For protein quantification, only unique peptides were taken into consideration. For each label, protein abundance was defined as the average of the peptide quantification values belonging to the given protein, which is expected to better reflect the overall change at the protein level (in comparison to using the median values), due to the expected ionization efficiency differences among different peptides. Subsequently, the average values were calculated for cases and controls, and these values were log2 transformed. The ratio was calculated by following subtraction of the mean value obtained for case and controls, as in the case of label-free approach. As an alternative quantification strategy (referred as analysis 2), balanced quantification values were employed to calculate the peptide ratio. The latter was expressed as a ratio of quantifications values corresponding to pT2+ vs. pTa samples. Similarly, only unique peptides were considered for protein quantification. Protein ratio was calculated by the averaging of all quantifiable peptide ratios belonging to each protein and the ratio values obtained were subsequently log2 transformed.

### Statistical analysis

Statistical analysis was performed using SPSS Statistical Software (SPSS 17.0, IBM). For each quantification method, the p-value was calculated for the log2 transformed values by using independent sample t-test. In the case of the alternative quantification approach tested for the iTRAQ (analysis 2), the p-value was calculated based on the normal distribution of the ratios by using R programming language. Proteins with a p-value below 0.05 were considered as statistically significant. Pearson correlation and regression analysis was calculated in MedCalc Version 12.1.0.0 (Mariakerke, Belgium).

### Assessment of reliability of protein identification and differential expression

Validity of the received protein identification was assessed by comparison to expression data from urinary bladder and/ or bladder cancer tissue reported in the Human Protein Atlas (http://www.proteinatlas.org/ [30]), ProteomicsDB (http://www.proteomicsdb.org/ [31]) as well as transcriptomic resources (Bgee Database [32]). Credibility of the regulation trend (up-/down-regulated in pT2+ vs. pTa), as obtained from the proteomic analysis, was evaluated based on comparison with the mRNA microarray data (GSE3167 [33]) deposited in the Gene Expression Omnibus [34] as well as literature [35, 36]. The former transcriptomic data obtained for the pT2+ and pTa bladder cancer stages were analyzed by the GEO2R [37], a web tool enabling statistical analysis of the data. The information about analyzed samples as well as the output from the GEO2R is presented in S2 Table. The expression trend reported in proteomics was considered valid when agreement between the proteomic and microarray data was observed.

### Immunohistochemistry

Immunohistochemical evaluation of Annexin A6 expression (Annexin VI Antibody (N-19), polyclonal, anti-goat, Sc-1931, Santa Cruz, AB_630873 Antibody Registry ID, dilution 1:200) was performed on a tissue microarray containing 35 tissue samples (n = 11 non-cancerous bladder samples, n = 8 pTa tumors, n = 8 pT1 tumors, n = 8 pT2+ tumors). Further visualization was performed with diaminobenzidine according to the manufacturer instruction (ultraView Universal DAB Detection Kit) and subsequently sections were counterstained with hematoxylin. Quantification of the staining was performed with ImageJ software after application of color deconvolution [38]. Briefly, 5 images were acquired per section and 10 identical areas among the sections were selected for measurement. The optical density for the background was subtracted from all measurements.

## Results

**Fig 1:**
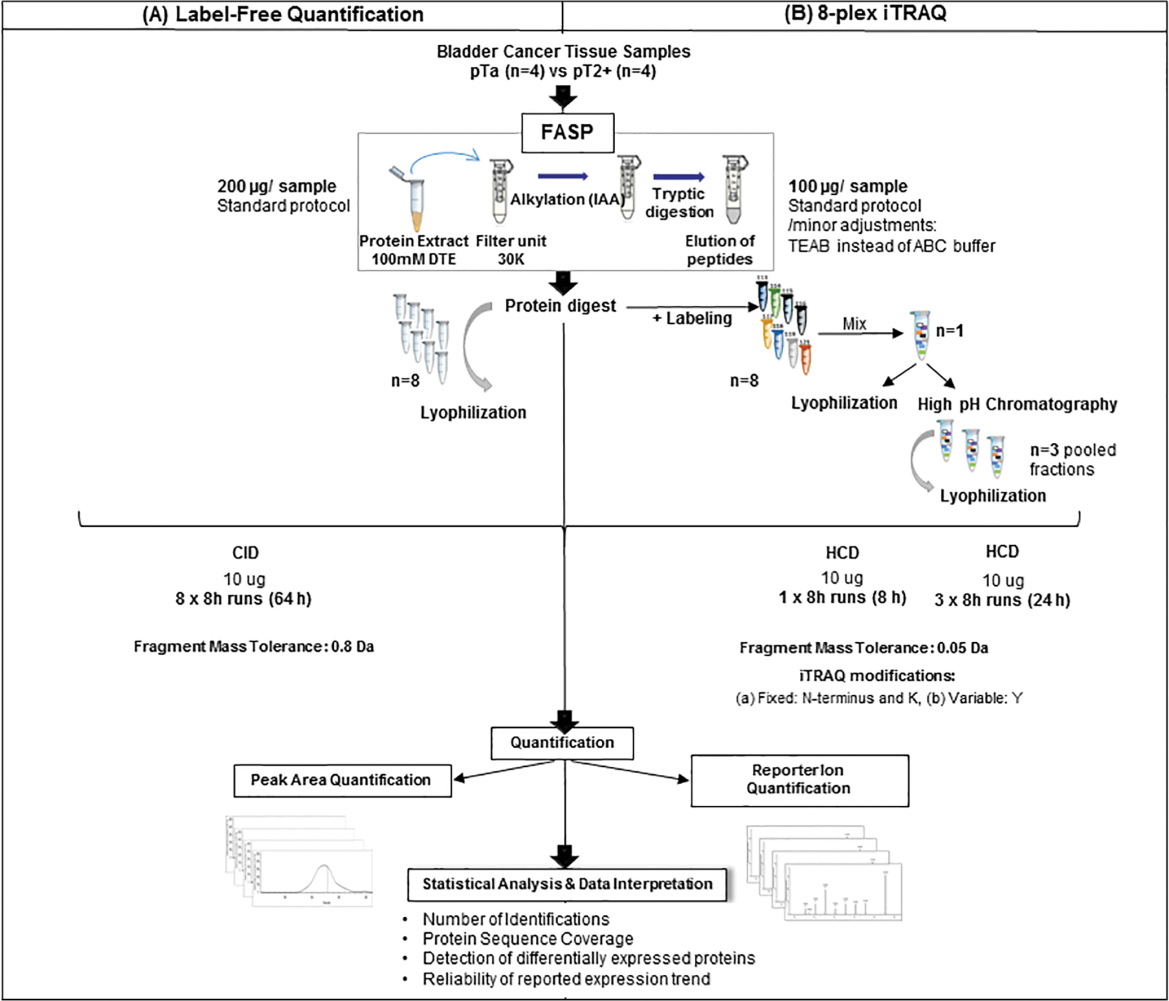
Experimental workflow. The applied workflow for sample preparation and data analysis for LFQ and iTRAQ quantification is graphically depicted.

Three experimental approaches were evaluated (label-free, unfractionated and fractionated iTRAQ) aiming to select the optimal strategy for determination of protein differential expression in highly complex samples employed in clinical proteomics (i.e. non-muscle invasive (pTa) in comparison to muscle-invasive (pT2+) bladder cancer). The workflow for sample preparation and data analysis is depicted in Fig 1. Aliquots of the same protein extracts were used in all cases and all samples were processed by FASP. The lysis buffer was selected based on preliminary experiments which showed its efficiency (in terms of protein recovery and reproducibility) for bladder tissue (data not shown). In the case of iTRAQ some minor modifications of the classical FASP protocol were necessary to ensure compatibility with the subsequent labeling, as suggested by the manufacturer and described earlier [39–41]. These include a reduction of the initial amount of protein processed by FASP (from 200 μg used for LFQ to 100 μg for iTRAQ), and substitution of the ammonium bicarbonate buffer with triethylammonium bicarbonate to avoid interference of the former with labeling via interactions with the iTRAQ reagents.

The impact of possible differences introduced by the sample preparation protocols was assessed based on the prevalence of defined modifications in both experiments. The analysis was conducted using Preview™ software [25] and the results were subsequently evaluated according to the percentage and the fraction of modified peptides (ratio of number of peptides containing modifications vs. peptides that could possibly contain modification). In general, a comparable percentage of modified peptides between both approaches (LFQ and iTRAQ) was obtained, as confirmed by regression analysis (S1 Fig). Most reported modifications were identical (S3 Table) and the frequency of appearance was generally low. For few modifications, there was a significant difference between the percentage of modified peptides in LFQ and iTRAQ including a) formation of Pyro-glu N-terminus, b) carbamylation of methionine, c) N-terminal acetylation of proteins, d) oxidation of methionine/ histidine/ tryptophan and e) carbamidomethylation artifacts.

Mass spectrometry and data analysis were performed according to optimized protocols for each approach, as described in Materials and Methods. In this way, we targeted to obtain unbiased and representative results for each approach.

### Protein identification and Quantification

To ensure validity of the findings, only consistently detected peptides (in at least 75% of samples of one group (pTa or pT2+) were considered in the LFQ approach. As indicated in Materials and Methods, peptides representing possible contaminations such as keratins were completely removed from the datasets and thus, the corresponding proteins were not taken into consideration in further analysis. Based on this threshold, an average number of 5184±550 peptide and 1113±78 protein identifications, including single peptide hits, were reported per LC-MS/MS run (Table 1). In total the LFQ approach enabled the identification of 6871 peptides corresponding to 1346 proteins. For unfractionated iTRAQ, a total number of 1859 peptides and 664 proteins were reported; pre-fractionation of the labeled peptides increased the identification rate for both, peptides (6099) and proteins (2064). However, in the two iTRAQ experiments approximately 49% of the reported proteins were represented by a single peptide only. The respective percentage for LFQ was 32%. Upon exclusion of proteins represented by a single-peptide, a total of 332, 1092 and 910 protein identifications are received in the unfractionated, fractionated iTRAQ and LFQ experiments, respectively. The lists of identified peptides and proteins per technique (including also single-peptide identifications) are presented in S4 Table.

**Table 1:** Overview of the number of peptides and the corresponding proteins as being identified in the individual MS-runs.

Method	Sample ID	# peptide groups	# protein groups
	3_pTa	5073	1096
	11_pTa	5269	1099
	16_pTa	5725	1185
	19_pTa	3931	937
**LFQ**	**Mean (SD)_pTa**	**5000 ± 763**	**1079 ± 103**
	9_pT2+	5360	1130
	12_pT2+	5112	1136
	15_pT2+	5516	1169
	17_pT2+	5485	1155
	**Mean (SD)_pT2+**	**5368 ± 184**	**1148 ± 18**
**iTRAQ**	10 μg	1859	664
	**fractionation**	6099	2064

**Fig 2:**
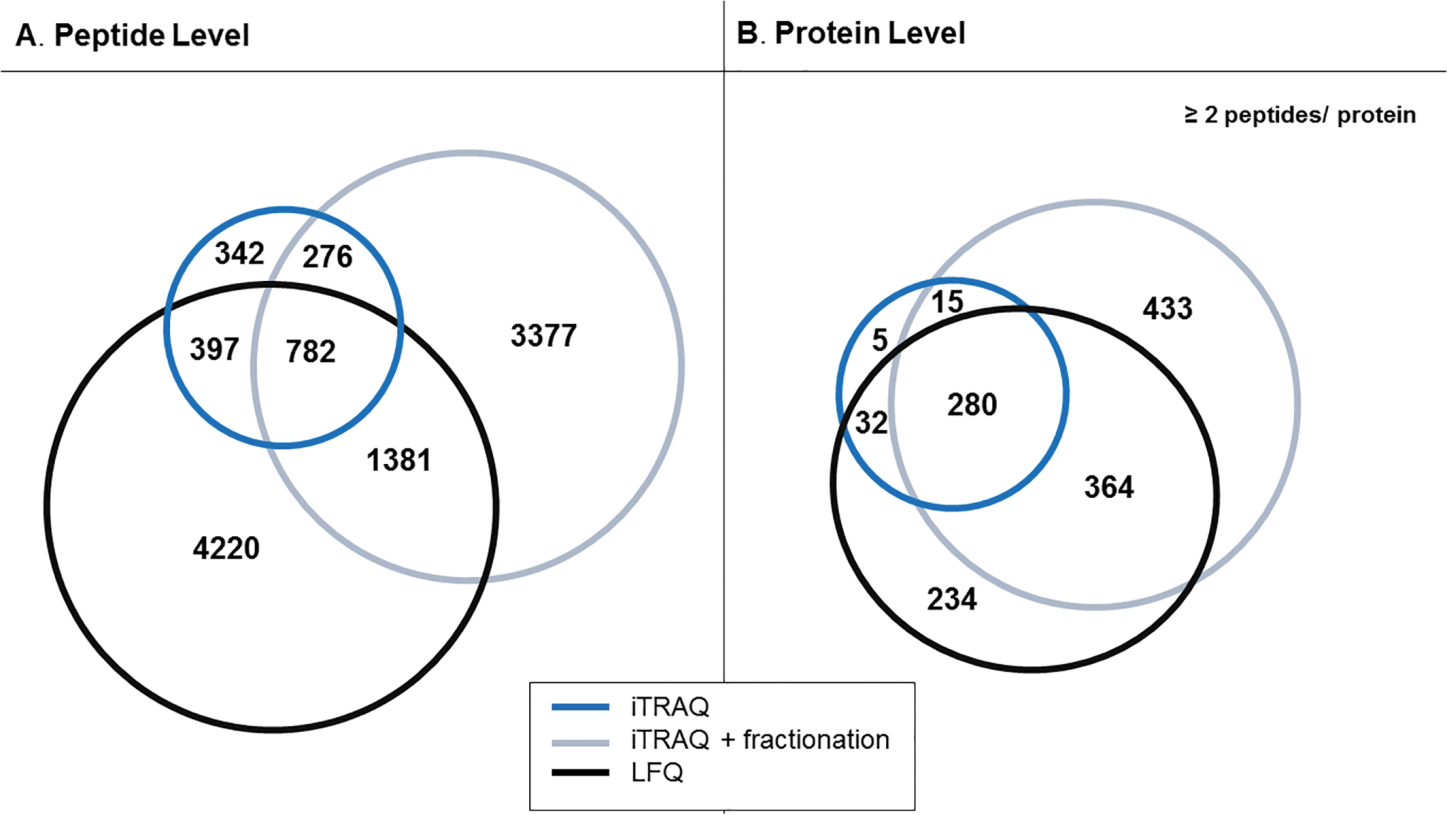
Comparison of peptide and protein identifications in iTRAQ and LFQ experiments. Venn diagrams representing the comparison of all identified peptides, without considering fixed/variable modifications (A), and proteins (B) from LFQ, fractionated/ unfractionated iTRAQ analysis.

The obtained data were subsequently compared at both peptide (Fig 2A) and protein levels (Fig 2B). For this analysis and to increase reliability of findings only multiple peptides (≥ 2) identifications were taken into consideration. As represented in the Venn diagram (Fig 2A), 782 peptides were reported in all three approaches, which corresponds to 42% and 13% of the total number of the peptides detected by the non-fractionated and fractionated iTRAQ respectively and 11% of peptides identified by the label-free approach. When comparing the data at the protein level, 280 proteins were found to be detected by all acquisition methods (Fig 2B). This overlap corresponds to 84% of the proteins identified by iTRAQ unfractionated, 26% for iTRAQ-fractionated and 31% for LFQ. When comparing the proteins exclusively detected per method, an approximately two fold higher number of uniquely identified proteins (433 IDs) was obtained for the pre-fractionated iTRAQ sample as compared to the LFQ (234 IDs); a limited number of proteins exclusively identified in the unfractionated samples was also observed (5 IDs). Of note, in the LFQ approach all identified peptides and proteins have a quantification value, whereas 76% (1441 out of 1859 peptides) and 81% (4918 out of 6099 peptides) of the peptides detected by the iTRAQ experiments, without and with fractionation, respectively, could be quantified, as per the iTRAQ restrictions (unique labeled peptides detected in all clinical samples with a percentage of the co-isolation interference below 30% are considered as quantifiable in iTRAQ). At the protein level the vast majority of the proteins without a quantification value were single-peptide identifications. Of the proteins identified with at least two peptides, the quantification was not possible for 6 out of 332 and 14 out of 1092 proteins in the case of unfractionated and pre-fractionated iTRAQ sample, respectively.

In the case of the label-based approach, two methods were tested to calculate the abundance at protein level and subsequently assess the protein ratio. Either by calculating the average peptide quantification values (separately for each label) assigned to the protein (analysis 1) or by employing the averages of the individual peptide ratios (analysis 2) (as described in detailed in Materials and Methods). To exemplify differences related to the assessment of protein abundance, the two methods were tested for the data obtained from the fractionated iTRAQ sample. The obtained results are presented in S4 Table. The methods give highly comparable results: the ratios for de-regulated proteins (as indicated by the primary analysis, analysis 1) as well as for whole dataset (including proteins identified with ≥2 peptides) were significantly correlated between both methods, with the Pearson correlation coefficient of 0.99 (p<0.0001, S2 Fig). Moreover, comparable numbers of significant changes (p<0.05) were reported using both methods (45 and 48 for analysis 1 and 2, respectively).

### Coverage of protein sequence

**Fig 3:**
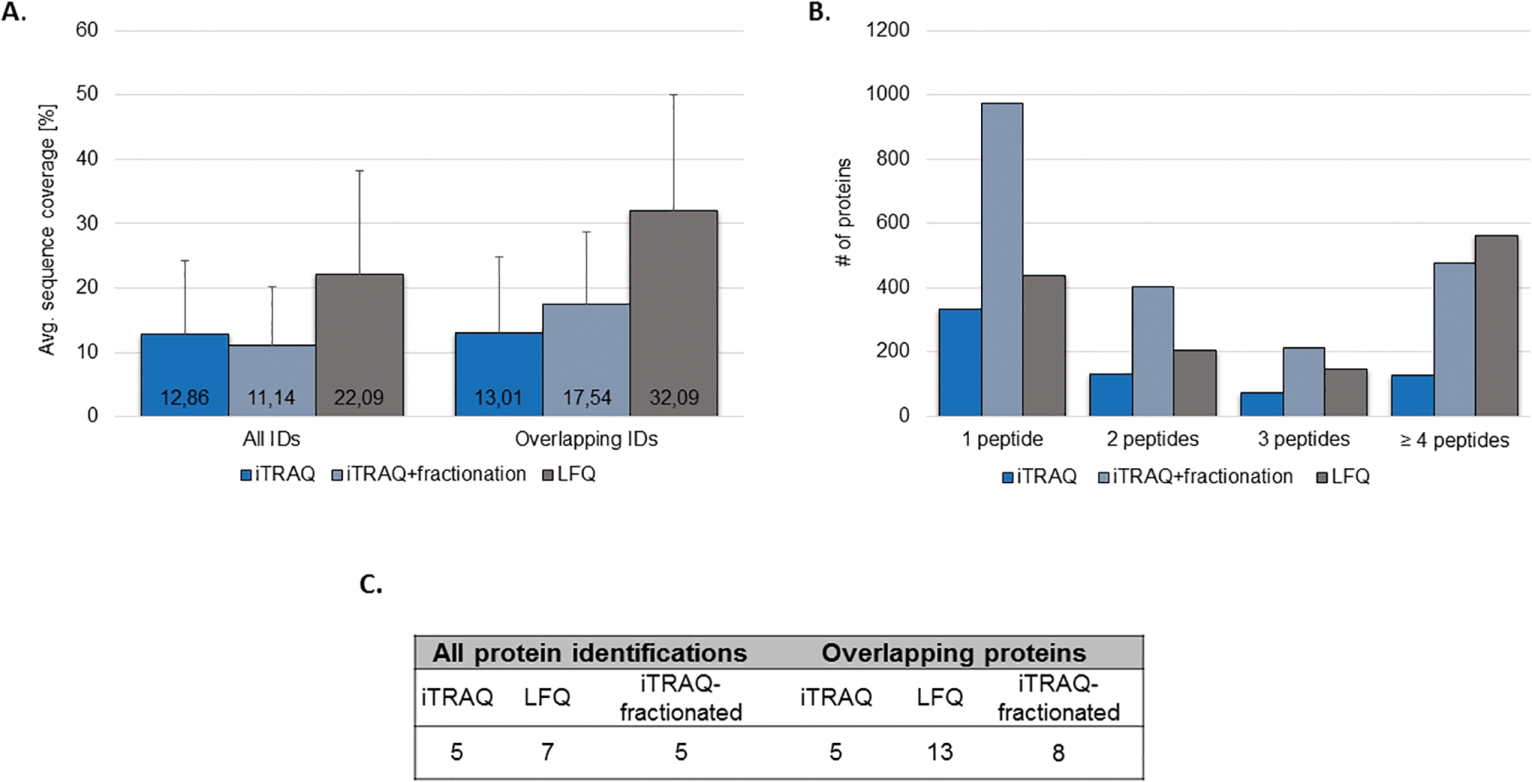
Evaluation of protein sequence coverage for LFQ and iTRAQ. Average protein sequence coverage was compared for all identified proteins per technique as well as for the overlapping identifications (A). The total number of identified proteins based on the particular number of peptides (B) and the average number of peptides per protein are also presented (C).

The protein sequence coverage was calculated for all the identified proteins per method and for the overlapping identifications between the three approaches (Fig 3A). Only multiple-peptide identifications (≥2 peptides) were considered. For iTRAQ experiments, a similar protein sequence coverage was reported for the unfractionated (13%) and fractionated (11%) sample. In the case of the LFQ, a significantly higher sequence coverage (22%, p<0.001 independent sample t-test) in comparison to both iTRAQ approaches was observed. This difference was even more pronounced when considering the overlapping identifications among different methods (32% vs. 13%/ 18% for LFQ and non-/ fractionated iTRAQ, respectively) (Fig 3A). The above observations are further supported by the evaluation of the average number of peptides per identified protein (Fig 3C) as well as a comparison of the number of proteins identified based on particular number of peptides (i.e. 1, 2, 3 and ≥ 4 peptides/ protein) per technique (Fig 3B). As shown, comparable numbers of single-peptide identifications were reported in LFQ (436 IDs) and iTRAQ-unfractionated (332 IDs). Fractionation of the labeled peptides increased the number of single peptide identifications substantially to 972 IDs. At the same time, fractionation also resulted in a higher number of multiple-peptides identifications (≥ 2 peptides) in comparison to the unfractionated iTRAQ (Fig 3B), but still at an overall lower average number of peptides per protein in comparison to LFQ (Fig 3C).

### Evaluation of differential expression

**Fig 4:**
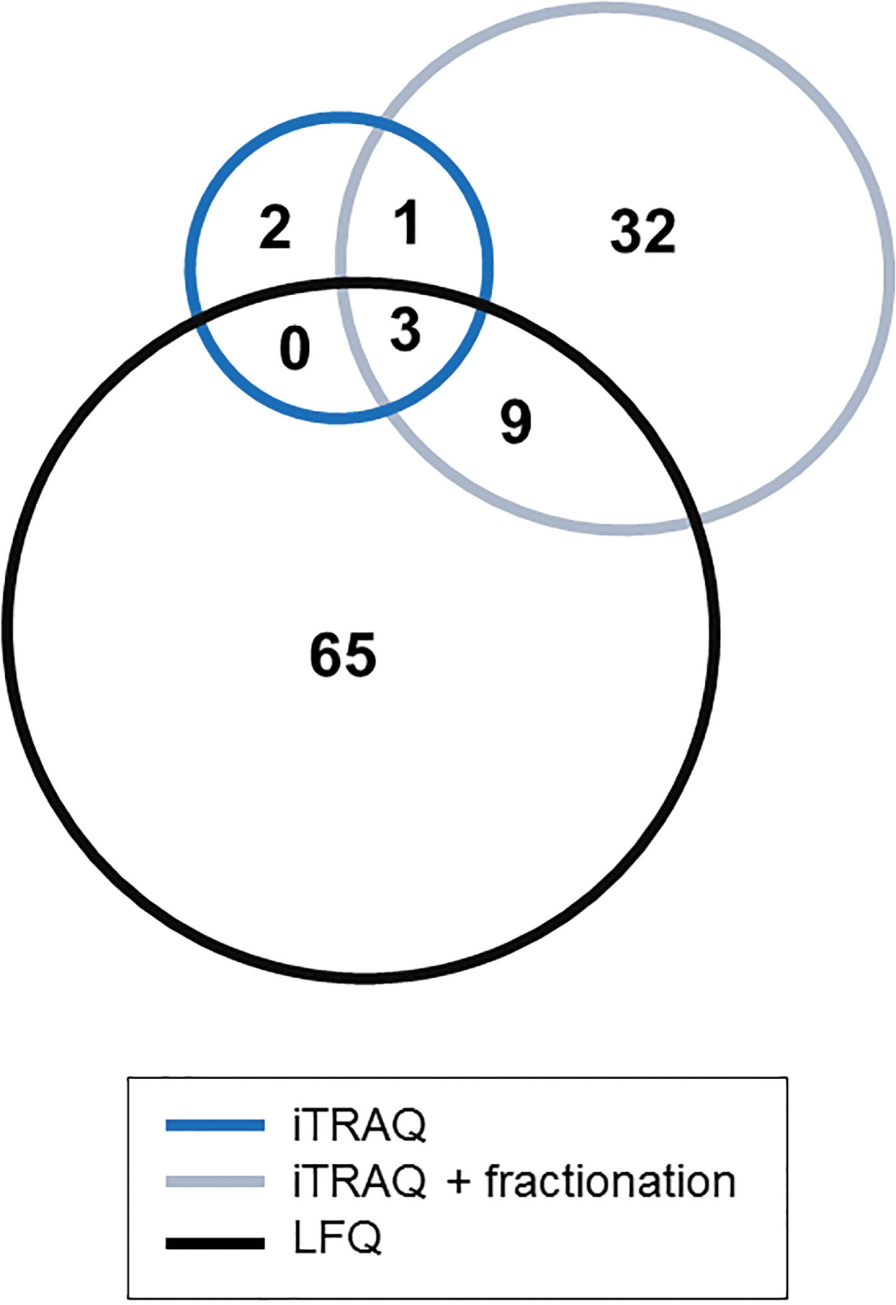
Comparison of differentially expressed proteins identified in both iTRAQ experiments and LFQ. Venn diagrams representing differentially expressed proteins found among the identified proteins after exclusion of single peptide hits.

Assessment of the relative protein abundance is based on the comparison of the quantification results of pTa (control) versus pT2+ (case) groups. Statistical analysis was used as a criterion to define the altered protein abundance. Thus, proteins with p-value < 0.05 were considered as being significantly changed in the case vs. control group. Additionally, the expression trend (up- or down-regulation in the case group) is represented by the ratio indicating the changes in the abundance between pT2+ over pTa BCa samples. Based on the statistical analysis (p<0.05), LFQ enabled identification of a higher number of differentially expressed proteins (77 proteins, identification based on at least 2 peptides), even in comparison to pre-fractionation of iTRAQ (45 proteins). The distribution of up- and down-regulated proteins is presented in Table 2. Three of these proteins were statistically significant in all three methods (Fig 4). On the other hand, 65 and 32 proteins were found to be statistically significant only in LFQ and fractionated iTRAQ samples, respectively (Fig 4). Of the former (65 proteins), as presented in Table 3, 49 proteins were identified by the other techniques but a significant difference in the relative abundance could not be detected. In the case of fractionated iTRAQ, the majority of proteins reported as uniquely differentially expressed were not identified by other methods.

**Table 2:** Comparison of number of differentially expressed proteins identified by LFQ and iTRAQ approaches.

Regulation Trend (≥ 2 peptides)	LFQ	iTRAQ	iTRAQ + fractionation
# Up-regulated	49	1	21
# Down-regulated	28	5	24
Total	77	6	45

Proteins with p-value < 0.05 were considered as differentially expressed. Based on the reported ratios (${\text{log}}_{2}\frac{case}{control}=Log_{2}Avg.Cases-Log_{2}Avg.Controls$) proteins were classified as up/ down regulated in pT2+ group (case group).

**Table 3:** Evaluation of the proteins with the altered abundance found as a unique based on the results obtained for three methods.

	LFQ	iTRAQ	iTRAQ + fractionation
Total number	65	2	32
Proteins identified in all three approaches	20	1	5
Proteins identified by two techniques	29	1	9
Exclusively identified	16	-	18

As indicated above, a very low number of identifications along with a low number of differentially expressed proteins were reported for the unfractionated iTRAQ approach in comparison to the other two techniques. The dataset obtained for the fractionated iTRAQ sample was considered as more favorable for the label-based approach and was included in further analysis.

In total, significantly altered levels of abundance (at least according to one quantification strategy i.e. LFQ and fractionated iTRAQ) were observed for 71 proteins (out of the overlapping 644 proteins detected by both methods).

**Fig 5:**
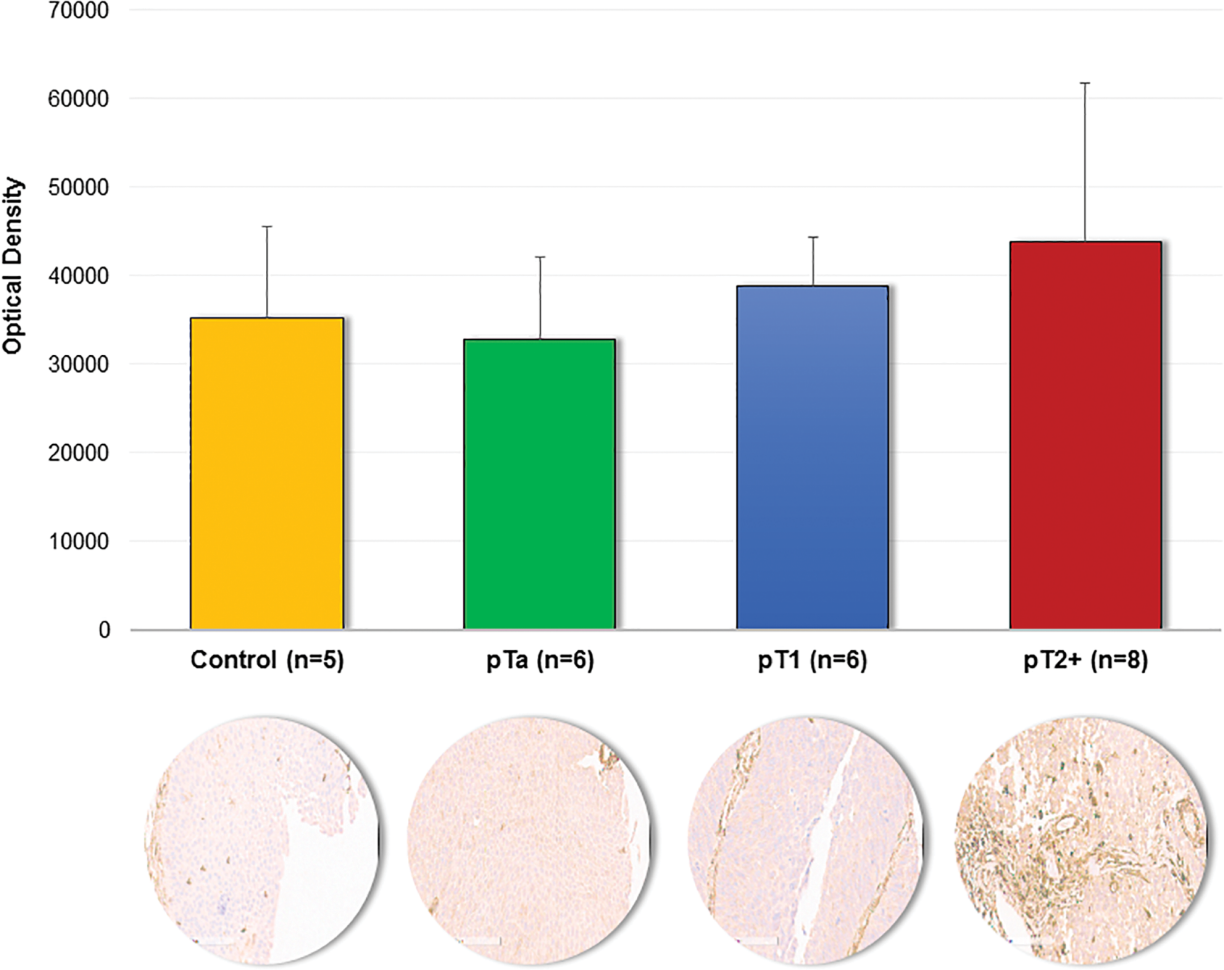
Immunohistochemical staining of Annexin A6. Quantification results obtained from non-cancerous tissue and bladder cancer tissues (pTa, pT1 and pT2+) along with the representative images of stained sections are presented. Quantification of the immunoreactivity was conducted by using Image J software followed by color deconvolution and background subtraction.

To further evaluate the consistency of the reported changes to these 71 proteins, S5 Table, the comparison of their regulation (up-/ down-regulated in pT2+) as reported by the different methods was performed. As shown (S5 Table), good consistency was observed: 66 proteins exhibit the same trend of expression, while 5 proteins appear to have inconsistent results (Table 4). Among the proteins exhibiting a consistent regulation, Annexin A6 was found to be increased using both proteomic approaches, but statistical significance was reached only for the case of LFQ. This expression trend was further confirmed by immunohistochemistry (Fig 5). For those 5 inconsistent quantification results observed between LFQ and iTRAQ, a comparison of the quantification values at the peptide level was conducted (Table 5). All of the proteins were quantified based on a comparable number of peptides. As presented in Table 5, the majority of the common peptides used for quantification were characterized by the opposite expression trend in the two methods.

**Table 4:** List of proteins with conflicting expression trend.

	iTRAQ + fractionation				Label-free			
Protein Name	#quantified peptides^a^	Log2Ratio	p-value	Regulation	# Peptides	Log2Ratio	p-value	Regulation
Actin-related protein 2/3 complex subunit 3	3	-0,25	**0.04** ^a^	down	2	0.37	0.47	up
Dolichyl-diphosphooligosaccharide—protein glycosyltransferase subunit 2	7	-0,06	0.58	down	6	1.23	**0.02** ^a^	up
KH domain-containing, RNA-binding, signal transduction-associated protein 1	2	-0.25	0.10	down	4	0.63	**0.02** ^a^	up
General vesicular transport factor p115	4	-0.10	0.57	down	3	0.52	**0.04** ^a^	up
Heterochromatin protein 1-binding protein 3	3	-0,30	**0.04** ^a^	down	6	0.72	0.17	up

Proteins that were found to be differentially expressed only according to one quantification method. Fold changes and p-values are reported.

^**a**^ Differentially expressed proteins with p-value <0.05.

**Table 5:** Comparison of the quantification results at the peptide and protein level for identifications with conflicting expression trends between fractionated iTRAQ and LFQ.

Protein/ Peptide		Peptide Log2 Ratio (pT2+ vs. pTa)		Protein Log2 Ratio (pT2+ vs. pTa)	
Protein Name	Peptide sequence used for quantification	iTRAQ + fractionation	LFQ	iTRAQ + fractionation	LFQ
	eASYSLIR	-0.24	0.03	**-0,30**	**0.72**
	mDAILTEAIk	-0,37	0.32		
**Heterochromatin protein 1-binding protein 3**	tIPSWATLSASQLAR	-0.30	0.41		
	SSAVDPEPQVK	-	1.31		
	LEDVLPLAFTR	-	0.24		
	GASGSFVVVQK	-	5.12		
	aYLQQLR	0.01	0.61	**-0.25**	**0.37**
**Actin-related protein 2/3 complex subunit 3**	lIGNMALLPIR	-0.43	-0.06		
	lIGNmALLPIR	-0.34	-		
	sIVEEIEDLVAR	0,21	2.46	**-0.06**	**1.23**
	eDQVIQLMNAIFSk	-0.36	-		
	fELDTSER	0.15	-		
	nFESLSEAFSVASAAAVLSHNR	-0.12	-		
**Dolichyl-diphosphooligosaccharide—protein glycosyltransferase subunit 2**	qEIQHLFR	0.09	-		
	yHVPVVVVPEGSASDTHEQAILR	-0.38	0.62		
	LQVTNVLSQPLTQATVK	-	0.33		
	ISTEVGITNVDLSTVDKDQSIAPK	-	1.78		
	NPILWNVADVVIK	-	3.65		
	YIANTVELR	0.06	3.15		
	KDDEENYLDLFSHK	-	0.39	**-0.25**	**0.63**
	ILGPQGNTIK	-	0.55		
**KH domain-containing, RNA-binding, signal transduction-associated protein 1**	DSLDPSFTHAMQLLTAEIEK	-	2.42		
	SGSMDPSGAHPSVR	0.06	0.68		
	qPPLPHR	-0.56	-		
	SSQTSGTNEQSSAIVSAR	-	0.27	**-0.10**	**0.52**
	SQLNSQSVEITK	-	0.25		
**General vesicular transport factor p115**	NDGVLLLQALTR	-0,23	2.44		
	eQDLQLEELR	-0.47	-		
	qSEDLGSQFTEIFIk	0.31	-		
	vASSTLLDDRR	-0.00007	-		

Similarly to the calculation of the relative abundance at the protein level, the peptide ratio values were calculated based on the log-2 transformed average vales for cases (pT2+) and controls (pTa).

### Validity of protein differential expression

We next evaluated whether the proteins identified as differentially expressed were previously detected in normal and/or malignant urothelium using various proteomics resources i.e. Human Protein Atlas (HPA) [30], ProteomicsDB [31], and gene expression database [32] (S6 Table). The expression of almost all differentially expressed proteins (39 out of 45 in iTRAQ and 64 out of 77 in LFQ, respectively) have been confirmed in normal and/or tumorous urothelium in all data repositories.

In an effort to further investigate the validity of the proteomics results, we compared them to transcriptomics data [33, 35, 36] from comparison of BCa vs. normal tissue [35], high vs. low grade bladder cancer [36] and/or invasive (pT2+) vs. non-invasive (pTa) BCa (Gene Expression Omnibus ID: GSE3167 [33]) (S6 Table).

The summary of the obtained results for datasets derived from the fractionated iTRAQ and LFQ is presented in Table 6. Of the differentially expressed proteins detected by LFQ 44% (34/77) were also found to be differentially expressed at the mRNA level (S6 Table). In the case of significant changes according to fractionated iTRAQ, the expression trend of 15 out of 45 proteins (33%) was in agreement with the microarray data. As aforementioned, a comparison of the differentially expressed proteins revealed several proteins as being significant only according to one approach.

**Table 6:** Assessment of the validity of the differentially expressed proteins identified in proteomics experiments.

**Overlapping IDs**			
	Total	Similar expression trend with transcriptomics	Not conclusive
Significant only in LFQ	45	18	27
Significant only in iTRAQ	14	3	11
Significant in both metods	12	3	9
**Unique IDs**			
	Total	Similar expression trend with transcriptomics	Not conclusive
LFQ	20	13	7
iTRAQ	19	9	10

The validity of the findings was evaluated by comparing the observed expression trends in this proteomics experiment with several transcriptomic experiments [35, 36]. Comparison of the detected changes was performed for the differentially expressed proteins reported among overlapping identifications between iTRAQ and LFQ as well as for the proteins solely detected in one approach (unique IDs). Proteins exhibiting similar expression trend in transcriptomics are presented in the “similar expression trend” column. In the cases when the expression trend was not in accordance to mRNA expression level or the data were not available, the findings were classified as “not conclusive”.

Of proteins that were detected in both approaches, but found as differentially expressed only based on one approach, 18 out of 45 for LFQ and 3 out of 14 for iTRAQ were also found to be differentially expressed at the mRNA level (Table 6, Overlapping IDs). In the case of the identifications solely detected by one technique (20 for LFQ and 19 for iTRAQ), the differential expression of 13 proteins (LFQ) and 9 proteins (iTRAQ) was supported by the mRNA data (Table 6, Unique IDs).

## Discussion

Since the introduction of the iTRAQ labeling as a quantification strategy for shotgun proteomics [42], several studies have been published aiming at the comparative analysis of label-free and iTRAQ performance [12–16]. Considering the advantages of multiplexing, MS analytical time and the total cost of the experiments is relatively lower in the iTRAQ analysis as compared to the LFQ. Additionally, since all the samples are measured simultaneously in a single MS run, the inter-run variations of the protein identification and quantification caused by the data-dependent acquisition do not exist. Reports presented in the past focused on the detailed “technical” characterizations of the quantification strategies applied in proteomics (including reproducibility, accuracy and precision) [12–16]. To the best of our knowledge a comparison of LFQ and iTRAQ as applied in the investigation of complex clinical samples has not yet been presented, especially in the context of the detection of the differentially expressed proteins.

We selected bladder cancer tissue specimens from the non-invasive (pTa) and muscle-invasive disease (pT2+) as a prototypic model for comparing the ability of iTRAQ and LFQ to efficiently detect differentially expressed proteins. In addition to the single LC-MS/MS analysis of the iTRAQ sample, peptide fractionation prior to LC-MS/MS analysis by using a high pH chromatography was also tested. All experiments were conducted using optimized protocols per technique as reported in the literature, to enable unbiased comparison of the two approaches. However, in order to minimize potential influence of biological and analytical variability on the quantification results, we applied, as far as possible, comparable sample processing strategies. Specifically, the same tissue extracts were employed in all cases generated using a protocol (FASP) for bladder tissue optimized in our laboratory. The FASP approach was selected to enhance both homogenization and protein solubilization process in bladder tumor specimens. The analyzed tissue specimens are considered difficult to be homogenized, therefore application of buffers containing high concentration of strong detergents (as in the case of FASP lysis buffer) is advisable. Additionally, application of the same approach for sample preparation and processing is crucial to avoid introducing any analytical bias related to variability of starting material. Even though the FASP method may not be considered optimal for iTRAQ analysis, adaptations were made according to existing literature ([39–41] also described in Materials and Methods). The small difference in the amount of protein initially processed by FASP (200 μg/ sample in LFQ and 100 μg/ sample in iTRAQ) likely does not affect the obtained results, since the same amount of protein was finally loaded onto the LC-MS/MS. The lower starting material in the case of iTRAQ was selected as one vial of the reagent can label between 20 and 100 μg of protein digest. We avoided acetone precipitation, as this can introduce additional variability and peptide modifications [43]. To assess whether the utilization of different protocols could introduce unanticipated modifications, we assessed the presence of known modifications using the Preview™ software [25]. The identity and the prevalence of vast majority of known modifications was similar in the iTRAQ and the LFQ approach (S1 Fig, S3 Table). This indicates that differences in the sample processing likely did not affect the obtained results. Following tissue processing, all subsequent steps, including proteins digestion and mass spectrometry analysis were conducted according to optimized protocols per LFQ and iTRAQ as described in the literature, in order to obtain representative results per technique.

The pivotal goal of the study was an evaluation of both quantification approaches, label-based and label-free, particularly focusing on the number and credibility of the identified differentially abundant proteins. To address this objective, sequence and proteome coverage as well as the capability of both techniques to detect the significantly altered proteins and, more importantly, the credibility of the identified changes were evaluated. This latter assessment was based on the comparison with existing expression data at the mRNA [32] and protein levels. The later included data deposited in Human Protein Atlas ([30], http://www.proteinatlas.org/) and ProteomicsDB ([31], http://www.proteomicsdb.org/) as well as relevant scientific literature [35, 36]. Since not only protein identification, but also the quantification process might be uncertain for single-peptide hits, we decided to assess the performance for all three acquisition methods based on proteins represented by at least 2 peptides identified.

### Comparison of proteome coverage

In our study, analysis of bladder tumor samples revealed a more than 2 times higher number of multiple-peptides based protein identifications in the label-free (910 IDs), than in the iTRAQ approach (332 IDs). However, this difference appears to be overcome when labeled peptides are pre-fractionated (1092 IDs). Along the same line, Patel et al. [13] reported a comparable number of proteins identified by both approaches during proteomics analysis of bacterium *Methylocella silverstris* (384 and 425 proteins were identified in iTRAQ and LFQ, respectively). In this report, the authors applied an additional peptide fractionation step prior to the MS analysis of iTRAQ samples [13]. In iTRAQ it was shown that, due to an increase in the average ion charge state, there is a significant reduction in the number of identifications [44]. Further, the iTRAQ 8-*plex* was reported to result in reduced protein annotation rate in comparison to the iTRAQ 4-*plex* [10]. During fragmentation, the loss of fragments of the label tag from precursor ions may occur, which causes some difficulties in the interpretation of the fragmentation spectra by the current search engines leading to the reduced peptide scoring.

We used the same set of tissue extracts for both experiments and sample processing was comparable (including homogenization and protein digestion), which minimized the biological and sampling variability on the number of detected proteins by LFQ and iTRAQ. The differences in fragmentation type of precursor ions, number of MS runs and MS run time are a consequence of utilizing standardized protocols for each strategy. Therefore, the collected data should reflect the optimized performance of each individual approach per se.

As indicated above, different fragmentation methods for data acquisition were applied: HCD in iTRAQ and CID in label-free experiments. For iTRAQ, HCD is mandatory for quantification, since the low masses of the reporter ions prohibit their detection in the ion trap. This was accompanied with the application of different thresholds for the fragment mass tolerance. However, this difference has been shown to not have a marked effect on the data [45]. To assess the possible influence of fragmentation strategy on the observed differences in the number of identified proteins, one of the selected tissue extracts from the label-free analysis was analyzed in duplicate under both experimental conditions i.e. CID and HCD fragmentation. This approach resulted in comparable number of total protein identifications (2270 and 2564 for CID and HCD, respectively) (S7 Table). Another explanation of the lower number of proteins identified in iTRAQ is related to the number of MS runs conducted per experiment. Since data-dependent acquisition is, to some extent, a stochastic process, the number of conducted MS runs in an experiment has an impact on the total number of identified peptides/ proteins. In the case of the LC-MS/MS analysis, consistently detected peptides (minimum frequency of 75% in one group) from 8 runs contribute to the total number of identified proteins. On the other hand, due to the multiplex character of the iTRAQ approach, all samples are analyzed in a single MS run. Performing duplicate runs of the iTRAQ sample (but in lower quantity than used for the presented results, e.g. 6 μg) leads to a slight increase in the total number of the identified proteins from 707/ 663 (for 1st / 2nd run) to 861 (S8 Table). Nonetheless, the number of identifications is comparable with the results obtained when 10 μg of protein was analyzed in a single run (664 proteins).

Advantages of iTRAQ are reduced MS run time and the ability to analyze multiple samples in a single run. Even though multiplexing in the iTRAQ approach may reduce the cost of experiments (by an 8 fold reduction of the MS run time) as well as decreased the inter-run variability, the added value of these features appears to be limited. However, after application of the pre-fractionation step an improved identification rate is achieved, being similar to the results obtained for LFQ. At the same time an advantage of shorter MS analytical time is maintained. In addition, some complementarity of both approaches is demonstrated [17, 18].

Collectively, based on our results, application of the fractionation strategy prior MS run provides superior results over conventional iTRAQ, and matches the increased number of identification in LFQ in part brought about by the multiple MS runs.

### Evaluation of quantification strategies

The quantification of the LFQ results was based on the sum of precursor ion areas for all peptides belonging to a protein. In iTRAQ the intensity of reporter ions is utilized. If the ion cannot be assessed, quantification is not possible [13]. This inability to perform quantification was experienced in about 6% of all peptides identified. The impaired quantification efficiency in iTRAQ might be related to insufficient peptide labeling [13]. However, in our experiments high labeling efficiency was observed. As an example, in the case of the fractionated iTRAQ sample, among the group of 1181 unquantifiable peptides (out of 6099), in 75 of these peptides the quantification value was not reported. High labeling efficiency is also supported by the results obtained using the Preview software (Protein Metrics Inc) [25]. The efficiency of the labeling is estimated based on percentage of labeled peptides vs. all possible labeling targets, as assessed based on the 100 most representative proteins in the dataset [25]. In our case, ∼98% of peptides containing K and ∼100% of peptides N-termini were detected as labeled. Comparable results were also obtained during the additional search in Proteome Discoverer, where the iTRAQ labels (N-termini, K) were set as variable modification. Over 99% of reported peptides carried the modification on N-terminal residue and/or lysine. In our study, the vast majority of unquantifiable peptides resulted from the criteria applied for inclusion of peptides for quantification including percentage of co-isolation interference (939 out of 1181 unquantifiable peptides) and peptide sequence uniqueness (167 out of 1181 peptides). Specifically, co-isolation (precursor mixing) is a well-known problem in iTRAQ, caused by selection of the precursor ions in a user-defined m/z window [28, 46, 47]. In this case, the co-isolation of other precursor ions may lead to contribution of non-related reporter ions to quantification. This has an impact on the accuracy of the quantification process. Sandberg et al. evaluated the impact of precursor mixing on the accuracy of the quantification [28]. For this purpose, a lysate of the breast cancer cell line (MCF7) was spiked with 57 standards and the effect of precursor mixing was investigated by co-analyzing iTRAQ (8-plex) and TMT (6-plex) labeled peptides. The bigger impact of the quantification accuracy was observed for the lower abundant proteins, which are particularly interesting as biomarker candidates. To reduce the effect of the precursor mixing on quantification accuracy, only spectra with the percentage of the co-isolation interference below 30% were included, yielding a good quantification accuracy [28].

Two different data analysis strategies were employed for the iTRAQ experiment. To keep similar criteria for identification of differentially expressed proteins between both, iTRAQ and LFQ, we have calculated the protein abundance based on averaging the quantification values from associated peptides. However, this approach may affect one of the strengths of the iTRAQ i.e. quantification at the level of MS/MS spectrum for each individual peptide. Considering this fact, we compared the conducted data analysis with the classical approach, where the protein abundance is calculated as an average of the peptide ratios belonging to the protein. Both methods enabled detection of comparable numbers of differentially expressed proteins, with the obtained ratios being highly consistent between both approaches, confirming that the obtained results were not affected by the selection of the quantification workflow. To further evaluate the quantification results, addition of the internal standard could be of substantial help and has been proven to be successfully applied by Sjödin et al during the evaluation of quantification results from various label-based and label-free techniques [14].

### Coverage of protein sequence

Our data demonstrate a general increase in protein sequence coverage in LFQ in comparison to both iTRAQ experiments, in agreement with previously published results [12, 13]. On the other hand, the limitation of MS-based approaches to identify low abundance proteins during global-proteomic analysis still exist [48]. Typically, difficulties in the detection of low abundance proteins are related to masking by proteins of higher abundance. During data-dependent acquisition, not all of the present ions are selected for fragmentation and usually, the ones excluded are the low-abundance peptides. As a result, the identification of low abundance proteins is often limited to single peptides. The additional pre-fractionation step resulted in an increased number of identified proteins with a single peptide. Hence, the application of the fractionation method may have a clear added value especially for the identification of low abundance proteins. However, the credibility of these findings has to be carefully assessed by the other techniques.

### Detection of differentially expressed proteins and their biological reliability

Differentially expressed proteins were defined based on the statistical analysis (p<0.05, independent sample t-test) to enable comparison based on the level of confidence. The fold change threshold was not taken into consideration, since the reported magnitude is not conclusive, particularly when comparing two quantification approaches which differ in performance. A meaningful comparison of the differential abundance based on the reported ratio appears questionable, since different thresholds are routinely applicable for each approach. On the other hand, even if a substantial change is observed, if it is not of statistical significance, then due to the low confidence should not be reported as a difference. However, when the interpretation of differentially expressed proteins in the specific biological context is of highest relevance, the assessment of the reliability of proteomic findings can be supported by volcano plots. The latter evaluation helps to eliminate apparent significant changes with low fold change, before the specific FDR is reached; thus the number of false positive changes will decrease in comparison to the analysis utilizing solely the level of significance as a criterion for defining differential expression.

A higher total number of changes was observed in the LFQ experiment (77 proteins) vs. both iTRAQ experiments (6 and 45 proteins were reported for the iTRAQ sample, without and with fractionation, respectively), which corresponds to 8% of total identifications in LFQ and 4% of quantified proteins in the fractionated iTRAQ sample. This trend was also observed previously by others (e.g. comparison of non-infected vs. infected with adenovirus human lung epithelial cells A549 [15] or *Chlamydomonas reinhardtii* sta6 and cw15 strains [16]).

In the data presented here, we observe that the highest fold change range of reported ratios was reported in label-free (-9.41 up to 9.33) and exceed the range observed in both iTRAQ experiments, being particularly prominent for the un-fractionated (-2.03 up to 1.88) vs. fractionated sample (-1.72 up to 2.60). This likely reflects underestimations of the ratios in iTRAQ due to isotopic impurities, sample complexity or efficiency of chromatography separation and is consistent with previous studies [16]. Overall, a good agreement in the measured relative abundance, as defined by the two strategies (e.g. up/ down regulated proteins according to the ratios), was observed. Preliminary results of immunohistochemical staining (IHC) for Annexin A6 confirm its increased expression in invasive tumors in line with the proteomics results (Fig 5).

Our analysis showed that among the reported significantly altered proteins (according to at least one method, LFQ or fractionated iTRAQ) only 5 proteins showed an inconsistency in the reported ratios. As reported in Tables 4 and 3 out of these 5 proteins were significantly differentially expressed only according to the LFQ analysis, and 2 proteins exhibited a significant change according to the fractionated iTRAQ. In general, in both approaches confidence in quantification results is supported by the comparable number of peptides contributing to protein quantification. On the other hand, since the confidence level of the differential expression for one of the methods is limited (p>0.05), the validity of the results from this particular method cannot be established. To evaluate the observed discrepancies, a literature search was conducted to examine concordance of our findings with published reports. However, the deregulation of these proteins has not been reported in the context of bladder cancer invasion (pT2+ vs. pTa). Thus, the reported changes in protein abundance has to be further verified in independent experiments such as Western Blotting or immunohistochemistry. Preliminary results of IHC staining for Annexin A6 confirm its increased expression in invasive tumors in line with the proteomic quantification results (Fig 5).

To further assess the reliability of the protein identification, the expression of the identified proteins in urothelium and bladder tumor was checked based on available data from proteomics and transcriptomics resources (S6 Table). The presence of almost all proteins in urothelial epithelium and/or bladder tumor was confirmed, thus supporting the reliability of the identification process. As presented, most of these proteins have been previously identified by other MS-based or immune-based experiments in the bladder (according to ProteomicsDb [31] or Human Protein Atlas [30]).

To further assess the credibility of the observed significant changes, a comparison of protein expression trends with transcriptomic studies [33, 36] for the invasive (pT2+) vs. non-invasive (pTa) and high versus low grade bladder cancer was performed. With the knowledge that some of these comparisons may not entirely correspond to changes at the protein level related to invasive versus non-invasive cancers, a higher percentage of confirmed differentially expressed proteins was observed for LFQ (34 out of 77 proteins, 44%) versus fractionated iTRAQ (15 out of 45 proteins, 33%). These data also showed that among the overlapping proteins, but found to be significant only according to one method, the 40% and 21% of the changes, as respectively indicated by LFQ and iTRAQ approach, were confirmed by transcriptomic data. Along the same line, a higher percentage of the confirmed changes among the proteins solely detected by one approach, was reported for LFQ (65%) compared to fractionated iTRAQ (47%) (Table 6). Consequently, based on the higher number of confirmed changes, the LFQ appears to have a better capability to detect differentially altered proteins in comparison to iTRAQ. However, since many of the changes could not be supported by the transcriptomic data, to make up for the risk of false associations received by both techniques, analysis of a higher number of samples and/or more stringent statistical analysis (e.g. adjustment for multiple testing) may be helpful.

## Conclusions

The presented work represents an unbiased comparison of two quantification approaches i.e. label-free and iTRAQ (unfractionated and fractionated analysis) in order to determine which technique is better suited for the detection of differential protein expression in clinical samples. In terms of the number of identified proteins, application of pre-fractionation of iTRAQ labeled peptides enables superior results over the conventional iTRAQ run; whereas the number of identified proteins was comparable to the LFQ. Based on the obtained results, the label free approach appears to be the preferred option, when the detection of differential expression is a main objective of the study. LFQ provides both a higher number and a higher percentage of differentially abundant proteins for which the change was also supported by the transcriptomics data. Additionally, the added value of LFQ over the iTRAQ is reflected by the more confident protein identification (higher protein sequence coverage). However, when time on the instrument or cost is a significant issue, iTRAQ may be the method of choice, when the pre-fractionation step is applied. Conclusively, label free quantitation may facilitate the characterization of the molecular mechanisms underlying pathological conditions. However, due to the possibility to detect false positive changes, an increase in the studied sample sizes, when possible application of stringent statistical criteria (e.g. adjustment for multiple testing) and further validation of findings are required.

## Supporting Information

S1 Fig Evaluation of the prevalence of modifications in label-based and label-free experiments using regression analysis (95% CI). Both analysis were performed based on average percentage of recognized modifications [number of peptides containing modifications/ number of peptides that could possibly contain modification * 100%]. For iTRAQ, results obtained from the fractionation experiment are shown. The average percentage of modified peptides was assessed based on the values obtained separately for each fraction; whereas for the label free experiment 3 randomly selected samples were evaluated. For the purpose of this analysis, iTRAQ derived modifications were excluded (Lysine and N-terminus set to a fixed modification of 304). The modifications exhibiting significantly different prevalence in iTRAQ and LFQ were highlighted.
(TIF)

S2 Fig Evaluation of protein quantification strategies for iTRAQ experiment. Pearson correlation analysis was performed for protein log2 ratios obtained for all quantified proteins (A) and significantly altered proteins, as indicated by analysis 1 (B). Only proteins identified with at least 2 peptides were included. In the case of analysis 1, protein abundance relied on the averages of peptide quantification values for each label and the protein ratio was calculated based on log2 transformed average values for cases and controls; whereas in the case of analysis 2, the protein ratio was calculated by averaging the ratios for individual peptides and the value was log2 transformed.
(TIF)

S1 Table Overview on the reporter ion isotopic distribution for iTRAQ (8-plex). (DOCX)

S2 Table Trascriptomics data for pT2+ vs. pTa generated by the GEO2R based on the data deposited in Gene Expression Omnibus (ID: GSE3167) [33]. (XLSX)

S3 Table Evaluation of the modification prevalence as calculated by Preview software. The percentage and the fraction (number of peptides containing modifications vs. number of peptides that could possibly contain modification) are reported for the iTRAQ (non-fractionated/ fractionated) and the LFQ experiments. In the case of the label-based approach supported with fractionation, the values are presented separately for each fraction); whereas for the label free experiment three randomly selected samples were evaluated.
(XLSX)

S4 Table List of identified peptides and proteins in both iTRAQ experiments and LFQ. Quantification values and expression trends as being obtained by using the label-free and iTRAQ approach are presented. In addition, in the case of the quantification of the results obtained after fractionation of iTRAQ sample, the ratios calculated based on the two protocols i.e. a) averaging of the peptide intensities belonging to protein, which was initially applied as well as b) alternative approach based on the calculation of protein ratios as an average of the peptide ratios.
(XLSX)

S5 Table Comparison of the expression trend for differentially expressed proteins found among overlapping identifications between LFQ and iTRAQ approach. (XLSX)

S6 Table Evaluation of reliability and relevance of identified changes in the iTRAQ and LFQ approach. The biological reliability of protein identifications was evaluated based on the data collected in proteomic (Human Protein Atlas [30], ProteomicsDB [31]) and gene expression (Bgee Database [32]) databases. Additionally, credibility of the detected changes was assessed through the comparison of mRNA expression in non-muscle invasive (pTa) vs. muscle invasive BC (pTa) [33], BCa cancer vs. normal tissue [35] and high grade vs. low grade BCa cancer [36]. (-) This protein-coding gene was not found as differentially expressed in presented studies. (‡) Proteins characterized by the similar expression trend with the transcriptomic study.
(XLSX)

S7 Table Evaluation of the number of proteins identified by using various fragmentation methods (HCD and CID). Results represent the total number of protein identifications from duplicate runs for selected sample. The presented lists were exported from Proteome Discoverer using following peptide filters: high confidence, ΔM<5ppm and rank 1. Further evaluation, apart exclusion of keratins, was not anticipated for the purpose of this comparison.
(XLSX)

S8 Table List of proteins identified in duplicate runs of the unfractionated iTRAQ sample. Common and unique proteins for each run are presented. The presented list of peptides were exported from Proteome Discoverer using following peptide filters: high confidence, ΔM<5ppm and rank 1. Further evaluation, apart exclusion of keratins, was not anticipated for the purpose of this comparison.
(XLSX)

## References

[1] Ong SE, Mann M. Mass spectrometry-based proteomics turns quantitative. Nat Chem Biol. 2005;1:252–262. 16408053 10.1038/nchembio736

[2] Ralhan R, Desouza LV, Matta A, Chandra Tripathi S, Ghanny S, Datta Gupta S, et al Discovery and verification of head-and-neck cancer biomarkers by differential protein expression analysis using iTRAQ labeling, multidimensional liquid chromatography, and tandem mass spectrometry. Mol Cell Proteomics. 2008;7:1162–1173. 10.1074/mcp.M700500-MCP200 18339795 PMC2424195

[3] Schiess R, Wollscheid B, Aebersold R. Targeted proteomic strategy for clinical biomarker discovery. Mol Oncol. 2009;3:33–44. 10.1016/j.molonc.2008.12.001 19383365 PMC2753590

[4] Eriksson H, Lengqvist J, Hedlund J, Uhlen K, Orre LM, Bjellqvist B, et al Quantitative membrane proteomics applying narrow range peptide isoelectric focusing for studies of small cell lung cancer resistance mechanisms. Proteomics. 2008;8:3008–3018. 10.1002/pmic.200800174 18654985

[5] Qattan AT, Mulvey C, Crawford M, Natale DA, Godovac-Zimmermann J. Quantitative organelle proteomics of MCF-7 breast cancer cells reveals multiple subcellular locations for proteins in cellular functional processes. J Proteome Res. 2010;9:495–508. 10.1021/pr9008332 19911851 PMC4261601

[6] Baker CL, Kettenbach AN, Loros JJ, Gerber SA, Dunlap JC. Quantitative proteomics reveals a dynamic interactome and phase-specific phosphorylation in the Neurospora circadian clock. Mol Cell. 2009;34:354–363. 10.1016/j.molcel.2009.04.023 19450533 PMC2711022

[7] Hubner NC, Bird AW, Cox J, Splettstoesser B, Bandilla P, Poser I, et al Quantitative proteomics combined with BAC TransgeneOmics reveals in vivo protein interactions. J Cell Biol. 2010;189:739–754. 10.1083/jcb.200911091 20479470 PMC2872919

[8] Husi H, Sanchez-Nino MD, Delles C, Mullen W, Vlahou A, Ortiz A, et al A combinatorial approach of Proteomics and Systems Biology in unravelling the mechanisms of acute kidney injury (AKI): involvement of NMDA receptor GRIN1 in murine AKI. BMC Syst Biol. 2013;7:110 10.1186/1752-0509-7-110 24172336 PMC3827826

[9] Bantscheff M, Schirle M, Sweetman G, Rick J, Kuster B. Quantitative mass spectrometry in proteomics: a critical review. Anal Bioanal Chem. 2007;389:1017–1031. 17668192 10.1007/s00216-007-1486-6

[10] Pottiez G, Wiederin J, Fox HS, Ciborowski P. Comparison of 4-plex to 8-plex iTRAQ quantitative measurements of proteins in human plasma samples. J Proteome Res. 2012;11:3774–3781. 10.1021/pr300414z 22594965 PMC3390908

[11] Werner T, Sweetman G, Savitski MF, Mathieson T, Bantscheff M, Savitski MM. Ion coalescence of neutron encoded TMT 10-plex reporter ions. Anal Chem. 2014;86:3594–3601. 10.1021/ac500140s 24579773

[12] Li Z, Adams RM, Chourey K, Hurst GB, Hettich RL, Pan C. Systematic comparison of label-free, metabolic labeling, and isobaric chemical labeling for quantitative proteomics on LTQ Orbitrap Velos. J Proteome Res. 2012;11:1582–1590. 10.1021/pr200748h 22188275

[13] Patel VJ, Thalassinos K, Slade SE, Connolly JB, Crombie A, Murrell JC, et al A comparison of labeling and label-free mass spectrometry-based proteomics approaches. J Proteome Res. 2009;8:3752–3759. 10.1021/pr900080y 19435289

[14] Sjodin MO, Wetterhall M, Kultima K, Artemenko K. Comparative study of label and label-free techniques using shotgun proteomics for relative protein quantification. J Chromatogr B Analyt Technol Biomed Life Sci. 2013;928:83–92. 10.1016/j.jchromb.2013.03.027 23608324

[15] Trinh HV, Grossmann J, Gehrig P, Roschitzki B, Schlapbach R, Greber UF, et al iTRAQ-Based and Label-Free Proteomics Approaches for Studies of Human Adenovirus Infections. Int J Proteomics. 2013;2013:581862 10.1155/2013/581862 23555056 PMC3608280

[16] Wang H, Alvarez S, Hicks LM. Comprehensive comparison of iTRAQ and label-free LC-based quantitative proteomics approaches using two Chlamydomonas reinhardtii strains of interest for biofuels engineering. J Proteome Res. 2012;11:487–501. 10.1021/pr2008225 22059437

[17] Jain R, Kulkarni P, Dhali S, Rapole S, Srivastava S. Quantitative proteomic analysis of global effect of LLL12 on U87 cell's proteome: An insight into the molecular mechanism of LLL12. J Proteomics. 2015;113:127–142. 10.1016/j.jprot.2014.09.020 25286751

[18] Sharma S, Ray S, Moiyadi A, Sridhar E, Srivastava S. Quantitative proteomic analysis of meningiomas for the identification of surrogate protein markers. Sci Rep. 2014;4:7140 10.1038/srep07140 25413266 PMC5382771

[19] Sobin L, Gospodarowicz K, Wittekind C. TNM Classification of Malignant Tumours, 7th edition UICC International Union Against Cancer Wiley-Blackwell 2009.

[20] Wisniewski JR, Zougman A, Nagaraj N, Mann M. Universal sample preparation method for proteome analysis. Nat Methods. 2009;6:359–362. 10.1038/nmeth.1322 19377485

[21] Bairoch A, Apweiler R. The SWISS-PROT protein sequence database and its supplement TrEMBL in 2000. Nucleic Acids Res. 2000;28:45–48. 10592178 10.1093/nar/28.1.45 PMC102476

[22] UniProt C. UniProt: a hub for protein information. Nucleic Acids Res. 2015;43:D204–212. 10.1093/nar/gku989 25348405 PMC4384041

[23] Eng JK, McCormack AL, Yates JR. An approach to correlate tandem mass spectral data of peptides with amino acid sequences in a protein database. J Am Soc Mass Spectrom. 1994;5:976–989. 10.1016/1044-0305(94)80016-2 24226387

[24] Kall L, Canterbury JD, Weston J, Noble WS, MacCoss MJ. Semi-supervised learning for peptide identification from shotgun proteomics datasets. Nat Methods. 2007;4:923–925. 17952086 10.1038/nmeth1113

[25] Kil YJ, Becker C, Sandoval W, Goldberg D, Bern M. Preview: a program for surveying shotgun proteomics tandem mass spectrometry data. Anal Chem. 2011;83:5259–5267. 10.1021/ac200609a 21619057 PMC3134881

[26] Vizcaino JA, Cote RG, Csordas A, Dianes JA, Fabregat A, Foster JM, et al The PRoteomics IDEntifications (PRIDE) database and associated tools: status in 2013. Nucleic Acids Res. 2013;41:D1063–1069. 10.1093/nar/gks1262 23203882 PMC3531176

[27] Serang O, Noble W. A review of statistical methods for protein identification using tandem mass spectrometry. Stat Interface. 2012;5:3–20. 22833779 10.4310/sii.2012.v5.n1.a2 PMC3402235

[28] Sandberg A, Branca RM, Lehtio J, Forshed J. Quantitative accuracy in mass spectrometry based proteomics of complex samples: the impact of labeling and precursor interference. J Proteomics. 2014;96:133–144. 10.1016/j.jprot.2013.10.035 24211767

[29] Deutsch EW, Mendoza L, Shteynberg D, Farrah T, Lam H, Tasman N, et al A guided tour of the Trans-Proteomic Pipeline. Proteomics. 2010;10:1150–1159. 10.1002/pmic.200900375 20101611 PMC3017125

[30] Uhlen M, Bjorling E, Agaton C, Szigyarto CA, Amini B, Andersen E, et al A human protein atlas for normal and cancer tissues based on antibody proteomics. Mol Cell Proteomics. 2005;4:1920–1932. 16127175 10.1074/mcp.M500279-MCP200

[31] Wilhelm M, Schlegl J, Hahne H, Moghaddas Gholami A, Lieberenz M, Savitski MM, et al Mass-spectrometry-based draft of the human proteome. Nature. 2014;509:582–587. 10.1038/nature13319 24870543

[32] Bastian F, Parmentier G, Roux J, Moretti S, Laudet V. Bgee: Integrating and Comparing Heterogeneous Transcriptome Data Among Species In: Bairoch A, Cohen-Boulakia S, Froidevaux C, editors. Data Integration in the Life Sciences. Berlin Heidelberg: Spinger; 2008 p. 124–131.

[33] Dyrskjot L, Kruhoffer M, Thykjaer T, Marcussen N, Jensen JL, Moller K, et al Gene expression in the urinary bladder: a common carcinoma in situ gene expression signature exists disregarding histopathological classification. Cancer Res. 2004;64:4040–4048. 15173019 10.1158/0008-5472.CAN-03-3620

[34] Edgar R, Domrachev M, Lash AE. Gene Expression Omnibus: NCBI gene expression and hybridization array data repository. Nucleic Acids Res. 2002;30:207–210. 11752295 10.1093/nar/30.1.207 PMC99122

[35] Kawakami K, Enokida H, Tachiwada T, Gotanda T, Tsuneyoshi K, Kubo H, et al Identification of differentially expressed genes in human bladder cancer through genome-wide gene expression profiling. Oncol Rep. 2006;16:521–531. 16865252

[36] Liu Y, Noon AP, Aguiar Cabeza E, Shen J, Kuk C, Ilczynski C, et al Next-generation RNA Sequencing of Archival Formalin-fixed Paraffin-embedded Urothelial Bladder Cancer. Eur Urol. 2014;66:982–986. 10.1016/j.eururo.2014.07.045 25199720

[37] Barrett T, Wilhite SE, Ledoux P, Evangelista C, Kim IF, Tomashevsky M, et al NCBI GEO: archive for functional genomics data sets—update. Nucleic Acids Res. 2013;41:D991–995. 10.1093/nar/gks1193 23193258 PMC3531084

[38] Ruifrok AC, Johnston DA. Quantification of histochemical staining by color deconvolution. Anal Quant Cytol Histol. 2001;23:291–299. 11531144

[39] Abdallah C, Sergeant K, Guillier C, Dumas-Gaudot E, Leclercq CC, Renaut J. Optimization of iTRAQ labelling coupled to OFFGEL fractionation as a proteomic workflow to the analysis of microsomal proteins of Medicago truncatula roots. Proteome Sci. 2012;10:37 10.1186/1477-5956-10-37 22672774 PMC3442994

[40] McDowell GS, Gaun A, Steen H. iFASP: combining isobaric mass tagging with filter-aided sample preparation. J Proteome Res. 2013;12:3809–3812. 10.1021/pr400032m 23692318 PMC4416645

[41] Zhang SY, Li BY, Li XL, Cheng M, Cai Q, Yu F, et al Effects of phlorizin on diabetic retinopathy according to isobaric tags for relative and absolute quantification-based proteomics in db/db mice. Mol Vis. 2013;19:812–821. 23592918 PMC3626294

[42] Ross PL, Huang YN, Marchese JN, Williamson B, Parker K, Hattan S, et al Multiplexed protein quantitation in Saccharomyces cerevisiae using amine-reactive isobaric tagging reagents. Mol Cell Proteomics. 2004;3:1154–1169. 15385600 10.1074/mcp.M400129-MCP200

[43] Simpson DM, Beynon RJ. Acetone precipitation of proteins and the modification of peptides. J Proteome Res. 2010;9:444–450. 10.1021/pr900806x 20000691

[44] Thingholm TE, Palmisano G, Kjeldsen F, Larsen MR. Undesirable charge-enhancement of isobaric tagged phosphopeptides leads to reduced identification efficiency. J Proteome Res. 2010;9:4045–4052. 10.1021/pr100230q 20515019

[45] Pejchinovski M, Klein J, Ramirez-Torres A, Bitsika V, Mermelekas G, Vlahou A, et al Comparison of higher-energy collisional dissociation and collision-induced dissociation MS/MS sequencing methods for identification of naturally occurring peptides in human urine. Proteomics Clin Appl. 2015. 10.1002/prca.201400163 25821083

[46] Karp NA, Huber W, Sadowski PG, Charles PD, Hester SV, Lilley KS. Addressing accuracy and precision issues in iTRAQ quantitation. Mol Cell Proteomics. 2010;9:1885–1897. 10.1074/mcp.M900628-MCP200 20382981 PMC2938101

[47] Ow SY, Salim M, Noirel J, Evans C, Rehman I, Wright PC. iTRAQ underestimation in simple and complex mixtures: "the good, the bad and the ugly". J Proteome Res. 2009;8:5347–5355. 10.1021/pr900634c 19754192

[48] Drabovich AP, Diamandis EP. Combinatorial peptide libraries facilitate development of multiple reaction monitoring assays for low-abundance proteins. J Proteome Res. 2010;9:1236–1245. 10.1021/pr900729g 20070123

