# Correction: Comparative Analysis of Label-Free and 8-Plex iTRAQ Approach for Quantitative Tissue Proteomic Analysis

**DOI:** 10.1371/journal.pone.0139268

**Published:** 2015-10-01

**Authors:** The PLOS ONE Staff

Tables 1 and 5 are incorrectly aligned due to errors introduced in the production process. The publisher apologizes for the errors. Please see the corrected Tables 1 and 5 here.

**Table 1:** Overview of the number of peptides and the corresponding proteins as being identified in the individual MS-runs.

Method	Sample ID	# peptide groups	# protein groups
**LFQ**	3_pTa	5073	1096
	11_pTa	5269	1099
	16_pTa	5725	1185
	19_pTa	3931	937
	**Mean (SD)_pTa**	**5000 ± 763**	**1079 ± 103**
	9_pT2+	5360	1130
	12_pT2+	5112	1136
	15_pT2+	5516	1169
	17_pT2+	5485	1155
	**Mean (SD)_pT2+**	**5368 ± 184**	**1148 ± 18**
**iTRAQ**	10 μg	1859	664
	**fractionation**	6099	2064

**Table 5:** Comparison of the quantification results at the peptide and protein level for identifications with conflicting expression trends between fractionated iTRAQ and LFQ

Protein/ Peptide		Peptide Log2 Ratio (pT2+ vs. pTa)		Protein Log2 Ratio (pT2+ vs. pTa)	
Protein Name	Peptide sequence used for quantification	iTRAQ + fractionation	LFQ	iTRAQ + fractionation	LFQ
**Heterochromatin protein 1-binding protein 3**	eASYSLIR	-0.24	0.03	**-0,30**	**0.72**
	mDAILTEAIk	-0,37	0.32		
	tIPSWATLSASQLAR	-0.30	0.41		
	SSAVDPEPQVK	-	1.31		
	LEDVLPLAFTR	-	0.24		
	GASGSFVVVQK	-	5.12		
**Actin-related protein 2/3 complex subunit 3**	aYLQQLR	0.01	0.61	**-0.25**	**0.37**
	lIGNMALLPIR	-0.43	-0.06		
	lIGNmALLPIR	-0.34	-		
**Dolichyl-diphosphooligosaccharide—protein glycosyltransferase subunit 2**	sIVEEIEDLVAR	0,21	2.46	**-0.06**	**1.23**
	eDQVIQLMNAIFSk	-0.36	-		
	fELDTSER	0.15	-		
	nFESLSEAFSVASAAAVLSHNR	-0.12	-		
	qEIQHLFR	0.09	-		
	yHVPVVVVPEGSASDTHEQAILR	-0.38	0.62		
	LQVTNVLSQPLTQATVK	-	0.33		
	ISTEVGITNVDLSTVDKDQSIAPK	-	1.78		
	NPILWNVADVVIK	-	3.65		
	YIANTVELR	0.06	3.15		
**KH domain-containing, RNA-binding, signal transduction-associated protein 1**	KDDEENYLDLFSHK	-	0.39	**-0.25**	**0.63**
	ILGPQGNTIK	-	0.55		
	DSLDPSFTHAMQLLTAEIEK	-	2.42		
	SGSMDPSGAHPSVR	0.06	0.68		
	qPPLPHR	-0.56	-		
**General vesicular transport factor p115**	SSQTSGTNEQSSAIVSAR	-	0.27	**-0.10**	**0.52**
	SQLNSQSVEITK	-	0.25		
	NDGVLLLQALTR	-0,23	2.44		
	eQDLQLEELR	-0.47	-		
	qSEDLGSQFTEIFIk	0.31	-		
	vASSTLLDDRR	-0.00007	-		
