# Supplementary figures and images for: Comparative Analysis of Label-Free and 8-Plex iTRAQ Approach for Quantitative Tissue Proteomic Analysis
Source: PLoS One. 2015 Sep 2;10(9):e0137048. doi: 10.1371/journal.pone.0137048 (PMC4557910; doi:10.1371/journal.pone.0137048)

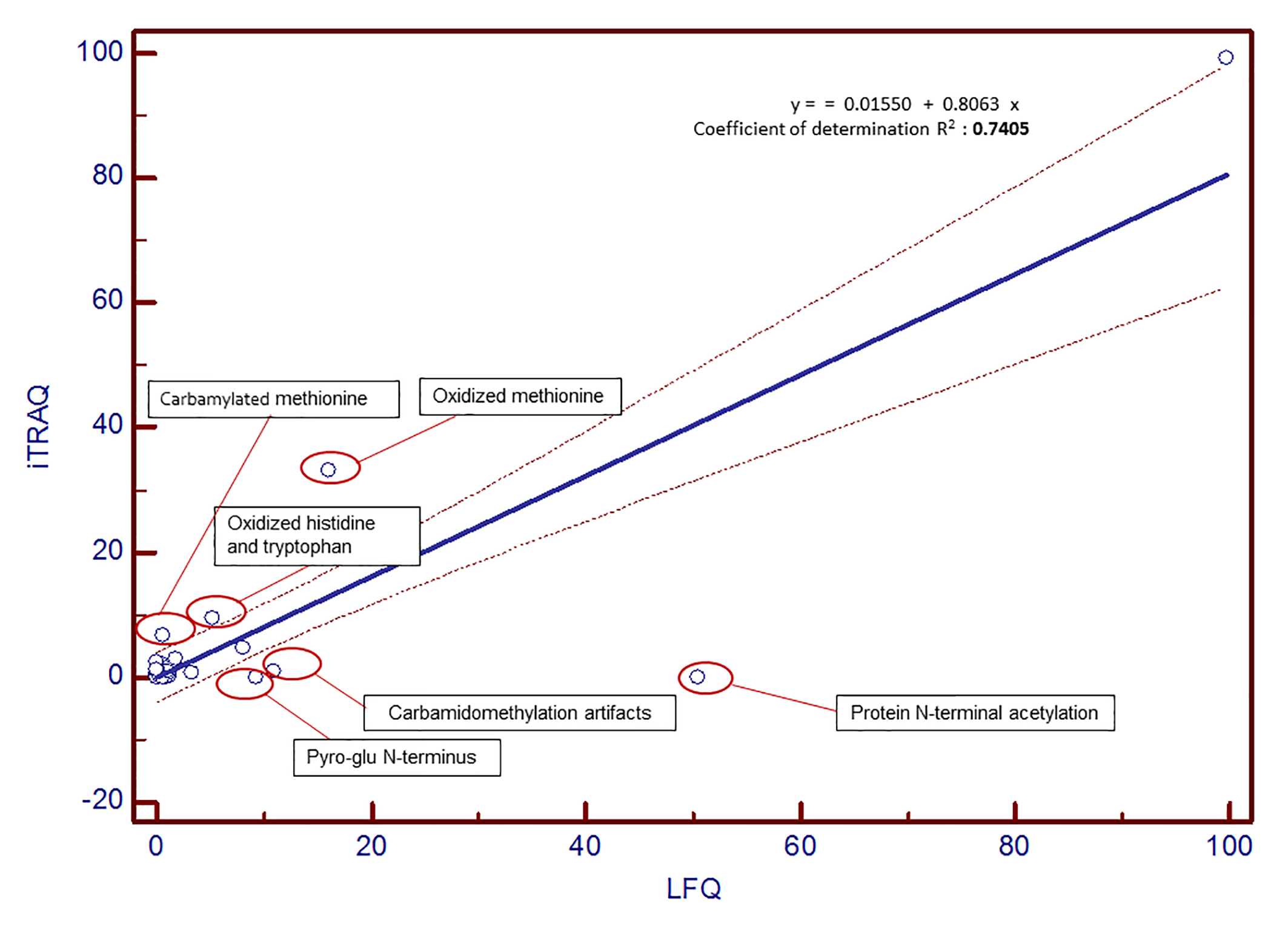

Supplement: S1 Fig — Both analysis were performed based on average percentage of recognized modifications [number of peptides containing modifications/ number of peptides that could possibly contain modification * 100%]. For iTRAQ, results obtained from the fractionation experiment are shown. The average percentage of modified peptides was assessed based on the values obtained separately for each fraction; whereas for the label free experiment 3 randomly selected samples were evaluated. For the purpose of this analysis, iTRAQ derived modifications were excluded (Lysine and N-terminus set to a fixed modification of 304). The modifications exhibiting significantly different prevalence in iTRAQ and LFQ were highlighted. (TIF) [file pone.0137048.s001.tif]

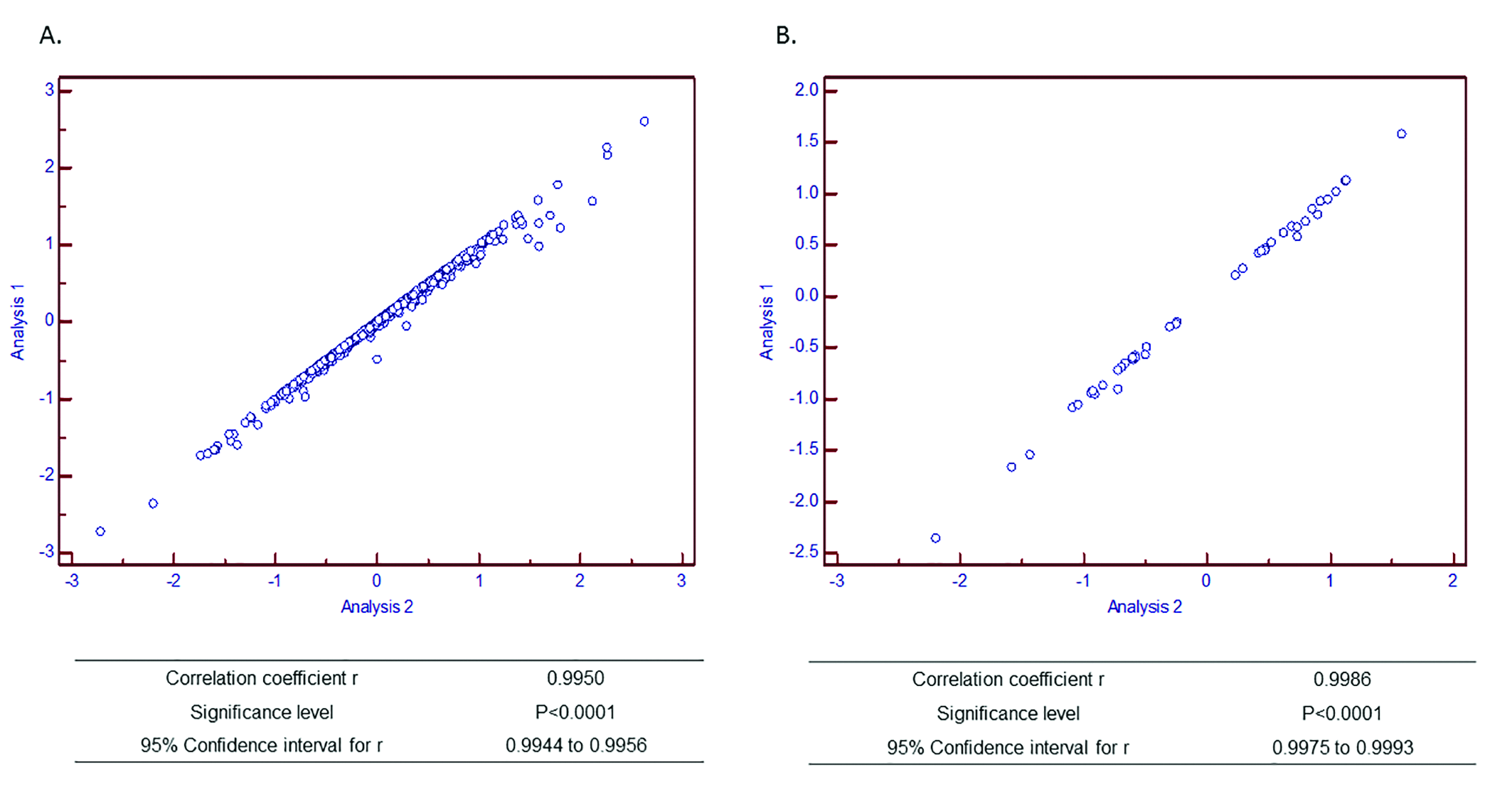

Supplement: S2 Fig — Pearson correlation analysis was performed for protein log2 ratios obtained for all quantified proteins (A) and significantly altered proteins, as indicated by analysis 1 (B). Only proteins identified with at least 2 peptides were included. In the case of analysis 1, protein abundance relied on the averages of peptide quantification values for each label and the protein ratio was calculated based on log2 transformed average values for cases and controls; whereas in the case of analysis 2, the protein ratio was calculated by averaging the ratios for individual peptides and the value was log2 transformed. (TIF) [file pone.0137048.s002.tif]
