# Supplementary material for: Comparative Analysis of Label-Free and 8-Plex iTRAQ Approach for Quantitative Tissue Proteomic Analysis
Source: PLoS One. 2015 Sep 2;10(9):e0137048. doi: 10.1371/journal.pone.0137048 (PMC4557910; doi:10.1371/journal.pone.0137048)
Supplement: S1 Table — (DOCX) [file pone.0137048.s003.docx]

|  | **-2(%)** | **-1(%)** | **0(%)** | **1(%)** | **2(%)** |
| --- | --- | --- | --- | --- | --- |
| **113** | 0 | 0 | 98,2 | 1,8 | 0 |
| **114** | 0 | 0,67 | 98,45 | 0,68 | 0,2 |
| **115** | 0 | 0,76 | 98,01 | 1,23 | 0 |
| **116** | 0 | 1,21 | 97,85 | 0,94 | 0 |
| **117** | 0 | 1,86 | 97,47 | 0,67 | 0 |
| **118** | 0 | 2,1 | 97,49 | 0,41 | 0 |
| **119** | 0 | 2,59 | 97,41 | 0 | 0 |
| **121** | 0 | 3,58 | 96,42 | 0 | 0 |
